# Digital health interventions for patient engagement: A systematic umbrella review

**DOI:** 10.1371/journal.pdig.0001785

**Published:** 2026-10-30

**Authors:** Usman Iqbal, Dumisani Enricho Nkhoma, Yankuba B. Manga, Afifa Tanweer, Syed Measum Haider Bokhari, Mochammad Fadjar Wibowo, Dina Nur Anggraini Ningrum, Annisa Ristya Rahmanti, Chih-Wei Huang, Hsuan-Chia Yang, Suleman Atique, Max Moldovan, Ayesha Humayun, Lutfan Lazuardi, Gulasekaran Rajaguru, Sharon Mickan, Leo Anthony Celi, Yu-Chuan Jack Li, Julie Redfern

**Affiliations:** 1 Institute for Evidence-Based Healthcare, Faculty of Health Sciences & Medicine, Bond University, Gold Coast, Queensland, Australia; 2 Evidence-Based Practice Professorial Unit (EBPPU), Gold Coast Hospital & Health Service (GCHHS), Gold Coast, Queensland, Australia; 3 Centre for Data Analytics, Bond Business School, Bond University, Gold Coast, Queensland, Australia; 4 School of Population Health, Faculty of Medicine and Health, University of New South Wales (UNSW), Sydney, New South Wales, Australia; 5 Master Program in Artificial Intelligence Applications and Management (AIAM), and Bachelor Program in Smart Sustainable Development and Management, International College of Sustainability Innovations, National Taipei University (NTPU), New Taipei city, Taiwan; 6 Department of Nutrition and Dietetics, School of Health Sciences, University of Management and Technology, Lahore, Pakistan; 7 Department of Medicine, Internal Medicine, Lausanne University Hospital and University of Lausanne, Lausanne, Switzerland; 8 Integrated Engineering Centre of Excellence (IECE), The University of Lahore, Lahore, Pakistan; 9 Faculty of Medicine, Public Health and Nursing, Universitas Gadjah Mada, Yogyakarta, Indonesia; 10 Master of Public Health Program, Faculty of Medicine, Universitas Negeri Semarang (UNNES), Semarang, Indonesia; 11 Department of Health Policy and Management, Faculty of Medicine, Public Health, and Nursing, Universitas Gadjah Mada, Yogyakarta, Indonesia; 12 Graduate Institute of Biomedical Informatics, College of Medical Science and Technology, Taipei Medical University, Taipei, Taiwan; 13 Department of Obstetrics and Gynaecology, Oslo University Hospital (OUS), Oslo, Norway; 14 Biometry Hub, Faculty of Sciences, Engineering and Technology, Adelaide University, Waite Campus, Adelaide, South Australia, Australia; 15 Department of Public Health and Community Medicine, Department of Medical Education (DME), SZMC Shaikh Khalifa Bin Zayed Al-Nahyan Medical College, Shaikh Zayed Postgraduate Medical Institute, Shaikh Zayed Medical Complex, Lahore, Pakistan; 16 Faculty of Health Sciences and Medicine, Bond University, Gold Coast, Queensland, Australia; 17 Laboratory for Computational Physiology, Massachusetts Institute of Technology, Cambridge, Massachusetts, United States of America; 18 Division of Pulmonary, Critical Care and Sleep Medicine, Beth Israel Deaconess Medical Center, Boston, Massachusetts, United States of America; 19 Department of Biostatistics, Harvard T. H. Chan School of Public Health, Boston, Massachusetts, United States of America; 20 International Centre for Health Information and Technology, College of Medical Science and Technology, Taipei Medical University, Taipei, Taiwan; 21 Department of Dermatology, Wan Fang Hospital, Taipei, Taiwan; 22 Research Centre for Artificial Intelligence in Medicine, Taipei Medical University, Taipei, Taiwan; 23 Sydney Nursing School, Faculty of Medicine and Health, The University of Sydney, Sydney, New South Wales, Australia; University of Technology Sydney, AUSTRALIA

## Abstract

Digital tools are increasingly integrated into patient engagement (PE) in chronic disease management, yet findings remain fragmented across populations, modalities, and outcome definitions. The aim of this umbrella review was to systematically review evidence on the effectiveness of Digital Health Interventions (DHIs) to enhance PE and related health outcomes. Six databases were searched for eligible studies published from inception to June 2025. Systematic reviews with meta-analyses of DHIs targeting engagement outcomes (e.g., self-management, adherence, activation) were included. Forty-two reviews (853 primary studies; ≈ 574,676 participants) met inclusion criteria, with most rated low-quality using AMSTAR-2. Meta-meta-analyses suggested DHIs could improve medication adherence (*SMD =* 0.65, 95% CI: 0.22, 1.08). Secondary outcomes also demonstrated potential for DHIs to improve quality of life (*SMD =* 0.26) and HbA1c (MD = −0.45%). Subgroup analyses indicated variation in effects across age groups and conditions for medication adherence and quality of life. Thus, digital health interventions may provide scalable, though moderate, improvements in patient engagement behaviours and selected clinical outcomes across diverse populations and conditions. Despite statistically significant pooled estimates for selected outcomes, due to the predominance of low-quality reviews, findings should be interpreted as exploratory evidence of effectiveness. Future implementation should be informed by higher-quality systematic reviews, longer-term primary studies, and additional evidence on patient activation and satisfaction.

## Author summary

Digital technologies such as mobile apps, telehealth, text messaging, patient portals, and wearable devices are increasingly used to help people take a more active role in managing long-term health conditions. We brought together evidence from 42 systematic reviews covering 853 primary studies and more than 574,000 participants. Digital interventions showed potential to improve medication adherence and to achieve small improvements in quality of life and blood sugar. However, the evidence is much less certain than these numerical results alone may suggest. Forty of the 42 reviews were rated as low or critically low in terms of methodological rigor, while some pooled results relied on only three reviews and results often differed substantially across studies. We therefore interpret the pooled estimates as signals of potential benefit rather than proof that a particular digital approach will work for every patient or health system. Future studies should use clearer definitions of patient engagement, report outcomes consistently, follow participants for longer, and examine which digital formats work best for different populations. These improvements are needed before strong clinical, or policy recommendations can be made.

## Introduction

In recent decades, patient engagement (PE) has emerged as a central determinant of healthcare quality, safety and system performance. The concept encompasses behaviours such as treatment adherence, self-management, and shared decision-making, all of which influence clinical outcomes and care experience [1–3]. Patient engagement is relevant across the care cascade, which involves diagnosis, disease management and the continuum of care. Different concepts become relevant at different stages of care, with health knowledge and patient activation emerging in the early stages, shared decision-making, self-management and adherence emerging during treatment and continuum of care (see Table 1) [4,5]. Global health systems increasingly prioritise PE as a strategic pillar for strengthening quality and sustainability, a stance reinforced by the World Health Organization’s 2023 World Patient Safety Day theme, “Engaging Patients for Patient Safety” [6]. Evidence consistently demonstrates that engaged patients experience better outcomes, greater adherence, and lower utilisation of healthcare resources [2,7]. Yet, sustaining high levels of engagement remains challenging, particularly among individuals with chronic diseases or limited access to continuous support.

**Table 1:** Description of the interrelationship amongst patient engagement constructs across the patient care pathway.

No.	Construct	Pre-contact	Initial contact	Post-contact	Continuum of Care
1	Health knowledge	Usually low. PE focus at this stage	Increased at this stage	Increased at this stage	Increased at this stage
2	Patient activation	Usually low.	Starts to increase. Shared decision-making is crucial here	Increased at this stage	High levels crucial for other constructs at this stage
3	Treatment adherence	x	x	Starts to emerge at this stage	High levels crucial for health outcomes
4	Self-management	x	x	Starts to emerge at this stage	Continues to increase, dependent on patient activation
5	Health outcomes	Risk factors	Symptomatic at this stage	Symptoms start to regress	Changes during mid & long-term outcomes start to emerge
6	Patient satisfaction	x	Service based	Service based	Satisfaction now a product of engagement process
7	Implementation outcomes	x	Utilisation & cost of service	Utilisation & cost of service	Utilisation & cost of service

Parallel to these challenges, the rapid digital transformation of healthcare has expanded opportunities to bolster PE. Mobile health (mHealth) applications, telemedicine, electronic health records (EHRs), wearable sensors, and online self-management tools now enable remote communication, personalised feedback, and real-time patient monitoring. Adoption of these modalities accelerated during the COVID-19 pandemic, highlighting their potential not only to bridge access gaps but also to strengthen patient involvement in care [8,9]. Furthermore, several other meta-analyses have shown that DHIs improve medication adherence and patient activation [10–12].

Nevertheless, the evidence base remains fragmented. Definitions of PE concepts vary widely, intervention modalities are heterogeneous, and outcome measurements lack standardisation [13]. Additionally, regulatory concerns, methodological variability, and equity considerations for DHIs further complicate interpretation and implementation [12–14]. Systematic umbrella reviews offer a rigorous approach to synthesising such distributed evidence across conditions and modalities [15,16]. This umbrella review therefore aimed to consolidate and quantitatively summarise the effectiveness of digital health interventions in improving patient engagement and associated clinical outcomes. The findings are intended to inform clinical practice, guide policy, and identify priorities for future research in digital health.

## Methods

This umbrella review adhered to PRISMA guidance [17] and followed methodological recommendations for umbrella reviews [18,19]. The protocol was prospectively registered with PROSPERO (CRD42024575453). The review was conducted as part of a project that also involved evidence gap map review. Study populations were categorised into adults, paediatric or mixed-age groups. Eligible interventions comprised telehealth, mHealth, eHealth, messaging systems, wearable devices, social media platforms, and gamified digital tools. Comparators included usual care, no intervention, waitlist controls, or non-digital equivalents. Primary outcomes included patient engagement measures such as self-management, medication and non-medication treatment adherence, patient activation, satisfaction, and health knowledge. Detailed definitions of primary outcomes and interventions are provided in Table A in S1 Appendix File. Secondary outcomes included physical activity, mental wellbeing, distress, depression, quality of life, and clinical indicators (blood pressure, HbA1c). Eligible study designs included systematic reviews with meta-analyses; reviews without meta-analysis were excluded.

Six databases were searched (Cochrane Library, PubMed, Web of Science, Scopus, EMBASE, and EBSCOHOST) from inception till June 2025 using keywords for “digital intervention,” “eHealth,” “mHealth,” “mobile health,” “telehealth,” “telemedicine,” “e-Health,” “digital health,” “internet intervention,” “online intervention,” “patient engagement,” “patient participation,” “patient involvement,” “patient activation,” “patient empowerment,” “self-management,” “adherence,” “patient satisfaction,” and MeSH terms for “Telemedicine,” “Mobile Applications,” “Health Information Systems,” “Patient Participation,” “Self-Care,” “Patient Compliance,” “Patient Satisfaction.” Boolean operators were applied, and detailed search strategies are provided in the Search Strategies per Database text of the S1 Appendix File.

Three independent reviewers performed study selection, completing deduplication and sequential screening of titles, abstracts, and full texts using Endnote 2025 [20]. Discrepancies were resolved through discussion with a fourth reviewer. Quality assessment was done independently using the AMSTAR-2 (A MeaSurement Tool to Assess systematic Reviews 2) [21]. Quality levels were categorised into high, moderate, low, or critically low based on the guidelines, with most reviews rated low or critically low due to limitations in protocol registration, inadequate reporting of reasons for study exclusion, lack of assessment of publication bias, and incomplete reporting. Corrected Covered Area (CCA) was calculated as ((N − r) ÷ [(c x r)-r] *100), where N is the total number of primary studies (including duplicates), r the number of unique primary studies, and c the number of included reviews. The CCA index was graded as slight (below 5%), moderate (between 6% and 10%), high (between 11 and 15%) and very high if over 15% [22].

Data extraction and synthesis were conducted manually using structured templates. Data extraction was conducted by AT, YM and MFW. One article had missing overall sample size data. However, since it was not involved in the meta-analysis, this did not affect the statistical analysis. For outcomes with at least three compatible reviews, defined as those reporting similar outcomes with similar effect measures, pooled effect sizes were computed, generally using random-effects models. Standardised mean differences (SMD) and mean differences (MD) were extracted where available. Heterogeneity was quantified using *I²* (with thresholds of 0–25% low, 26–75% moderate, > 75% high) [23]. Subgroup analyses were conducted for age group, condition, intervention type, and AMSTAR-2 rating when permitted by available data. Both meta-regression and publication bias assessment were considered but were not feasible due to insufficient pooled study counts. The review incorporated two deviations from the registered protocol, which involved performing a second search update and including a CCA calculation to align with best practices for umbrella reviews. All statistical analyses were conducted in R version 4.5.2 [24] with the meta (v8.2-1) [25] and metafor (v4.8-0) [26] packages.

## Results

### Study characteristics

Of the records identified through the database searches, 42 systematic reviews with quantitative data were found eligible for inclusion as shown in Fig 1. These reviews encompassed 853 primary studies, with 41 reviews reporting aggregate sample sizes totalling 574,676 participants and one review not reporting its sample size [27]. All reviews included randomised or non-randomised trials, with a small number (12%) also incorporating observational studies, and only one review including standalone qualitative research (see Table 2). The majority (83%) were published in or after 2020, reflecting a rapid recent expansion of digital health research. The overall CCA was 0.13%, which indicated low overlap across primary studies, suggesting a broad and largely non-redundant evidence base. Methodological appraisal using AMSTAR-2 revealed that 40 reviews (95%) were of low quality, with two rated critically low (Table B in S1 Appendix File).

**Table 2:** Characteristics of Included Reviews and Meta-Analyses on DHIs and Patient Engagement.

SR ID	Author (Name, Year, Country)	No. of Included Studies (k[N])	Types of Included Studies	Population details (Age group, conditions)	Interventions given (category & specific, period category)	Outcomes
1	Abdelraheem [12] 2024, Egypt	24 (24,137)	RCTs, nonrandomized, observational	Adults, > 18 years, (Cardiovascular, Diabetes, Cancers)	<12 months, mhealth, social media, ehealth	Patient Activation
2	Ampofo 2020 [28], Australia	12 (4205)	RCTs, observational	Adults, 41–74, Cardiovascular	Varying, telehealth	Adherence
3	Barakat 2019 [29], Australia	23 (2,754)	RCTs, nonrandomized	Adults, Mental Health	Varying, ehealth	Adherence, Service Utilisation,
4	Valentijn 2022 [30], Netherlands	56 (9,359)	RCTs	Adults, Musculoskeletal	<12 months, telehealth, mhealth	Self-Management, Disease Control, QoL
5	Verweel 2023 [31], Canada	17 (5,138)	RCTs	Adults, Various	<12 months, telehealth, mhealth, ehealth	Self-Management, Health Knowledge, Patient Activation
6	Akdemir 2024 [32], Australia	43 (8,622)	RCTs	Mix, Cancers	<12 months, mhealth, ehealth	Physical Activity, Adherence, Depression, Distress, Fatigue, QoL
7	Kamei 2020 [33], Japan	11 (655)	RCTs	Adults, Cardiovascular, Diabetes, Respiratory	<12 months, wearables	Adherence, Blood Glucose, Anthropometric Measures
8	Labarca 2021 [34], USA	16 (3,039)	RCTs	Adults, Obstructive Sleep Apnoea	<12 months, telehealth, ehealth	Adherence, Disease Control
9	Butler 2022 [35], Australia	15 (1,438)	RCTs, nonrandomized, observational	Children (<18 years), Musculoskeletal	<12 months, mhealth, wearables, ehealth	Self-Management, Physical Activity, Health Knowledge, QoL
10	Miao 2024 [36], China	21 (3,804)	RCTs	Adults, Cardiovascular	<12 months, telehealth, mhealth, messaging	Self-Management, Anthropometric Measures
11	Singleton 2022 [37], Australia	32 (4,790)	RCTs, nonrandomized	Mix, 8–80 years, Females, Cancers	<12 months, messaging, wearables, social media, ehealth	Self-Management, Adherence, Patient Satisfaction, Depression, Anxiety, Distress, Fatigue, QoL
12	Arsenijevic 2020 [38], Netherlands	27 (358,706)	RCTs, observational	Adults, > 18 years, Cardiovascular, Diabetes, Metabolic	<12 months, mhealth, ehealth	Adherence
13	McLean 2016 [39], UK (McLeanb)	5 (593)	RCTs	Adults, Respiratory	<12 months, mhealth, messaging, ehealth	Adherence, Disease Control, QoL
14	Groot 2023 [40], UK	19 (7,243)	RCTs, nonrandomized	Adults, Various	<12 months, mhealth, messaging, ehealth	Adherence, Well Being, Distress
15	Doumen 2022 [41], Belgium	45 (14,341)	RCTs, observational	Mix, Musculoskeletal	<12 months, messaging, wearables, ehealth	
16	Domhardt 2021 [42], Germany	26 (6,042)	RCTs	Adults, Mental Health	<12 months, mhealth, ehealth	Anxiety
17	Parsons 2021 [43], UK	10 (9,831)	RCTs, nonrandomized	Adults, pregnant women, Flu Immunisation	<12 months, mhealth, messaging, social media, ehealth	Service Utilisation
18	Liu 2022 [44], China	24 (9,634)	RCTs	Adults, Cardiovascular	<12 months, telehealth, mhealth, messaging, ehealth	Health Knowledge, QoL, Service Utilisation
19	Nkhoma 2021 [45], Taiwan	39 (6,861)	RCTs	Mix, 13–70 years, Diabetes	<12 months, mhealth, messaging, social media	Health Knowledge, Blood Glucose, QoL
20	Bunting 2021 [46], UK	6 (487)	RCTs	Adults, Musculoskeletal	<12 months, mhealth, messaging, ehealth	Adherence, Patient Activation
21	So 2018 [47], Hong Kong	7 (754)	RCTs	Adults, Diabetes	<12 months, telehealth, mhealth, messaging, ehealth	Patient Satisfaction, Blood Glucose
22	Esmaeili 2023 [48], Iran	12 (2,115)	RCTs	Adults, > 18 years, HIV	<12 months, telehealth, mhealth, messaging, ehealth	Adherence
23	Bevens 2022 [49], Australia	32 (2,950)	RCTs	Adults, Neurological	<12 months, mhealth, ehealth	
24	Alkhaldi 2016 [50], UK	14 (8774)	RCTs	Adults, > 18 years, Various	Varying, telehealth, messaging, wearables, ehealth	
25	Sin 2020 [51], UK	19 (1307)	RCTs	Adults, Mental Health	Varying, messaging, ehealth	Patient Satisfaction, Well Being
26	Aardoom 2020 [52], Netherlands	18 (5429)	RCTs	Adults, > 18 years, Obstructive Sleep Apnoea	<12 months, telehealth, ehealth	Disease Control
27	McLean 2015 [53], UK	8 (1110)	RCTs	Adults, > 18 years, Cardiovascular	Varying, mhealth, ehealth	Adherence, Disease Control, QoL
28	Pauley 2023 [54], Netherlands	47 (4958)	RCTs	Adults, > 18 years, Mental Health	<12 months, ehealth	Disease Control
29	Jeminiwa 2019 [55], USA	15 (13907)	RCTs	Mix, 3–65 years, Respiratory	<12 months, mhealth, messaging, social media, ehealth	Adherence
30	Tang 2020 [56], Australia	21 (2114)	RCTs	Adults, < 60 years, Transplantation (Surgical)	<12 months, telehealth, mhealth, wearables	Adherence, Patient Satisfaction, Disease Control, QoL
31	Vitija 2022 [57], UK	4 (502)	RCTs, nonrandomized	Children, 2–18, Mental Health, Neurodevelopmental	<12 months, telehealth, mhealth, messaging	Adherence
32	Oliveira 2023 [58], Portugal	15 (1619)	RCTs	Children, Mental Health	Varying, ehealth	Disease Control
33	Chan 2022 [59], New Zealand	40 (15207)	RCTs	Mix, Asthma	>12 months, messaging, wearables	Adherence, Disease Control, QoL
34	Kebede 2018 [60], Germany	21 (3787)	RCTs	Adults, Diabetes	<12 months, mhealth, messaging, ehealth	Self-Management, Risk Reduction, Disease Control
35	Seiler 2017 [61], USA	9 (1488)	RCTs	Adults, > 18 years, females (78%), Cancers	<12 months, ehealth	Depression, Distress, Fatigue, QoL
36	Khoong 2021 [62], USA	25 (6078)	RCTs, nonrandomized, mixed	Adults, mean 65 years, Cardiovascular	<12 months, mhealth, messaging	Self-Management, Adherence, Patient Satisfaction
37	Goldberg 2025 [27], Germany	17 (NR)	nonrandomized	Adults, > 18 years, Various	Varying, ehealth	
38	Osovskaya 2025 [63], UK	8 (1967)	RCTs, nonrandomized	Adults, Various	<12 months, ehealth	Patient Activation, Patient Satisfaction, Disease Control
39	Zhuang 2025 [64], Malaysia	17 (2027)	RCTs	Adults, > 60 years, Respiratory	<12 months, telehealth, mhealth, messaging, wearables, ehealth	Self-Management, Adherence, QoL, Service Utilisation
40	Ansari 2025 [65], UK	13 (1850)	RCTs	Adults, 55.8-66.7 years, Cardiovascular	<12 months, telehealth, mhealth, messaging, ehealth	Physical Activity, QoL, Service Utilisation
41	Cevasco 2024 [66], USA	14 (11059)	RCTs	Adults, > 18 years, females (65.8%), Various	>12 months, messaging	Adherence
42	Getie 2025 [67], Ethiopia	6 (3995)	RCTs	Adults, Diabetes	<12 months, telehealth, mhealth	Adherence, Disease Control

NB: NR: not reported, k; no. of studies, N: combined sample size of included studies, RCTs; randomised controlled trials, QoL; Quality of Life, Mix: a combination of adult and paediatric participants

**Fig 1:**
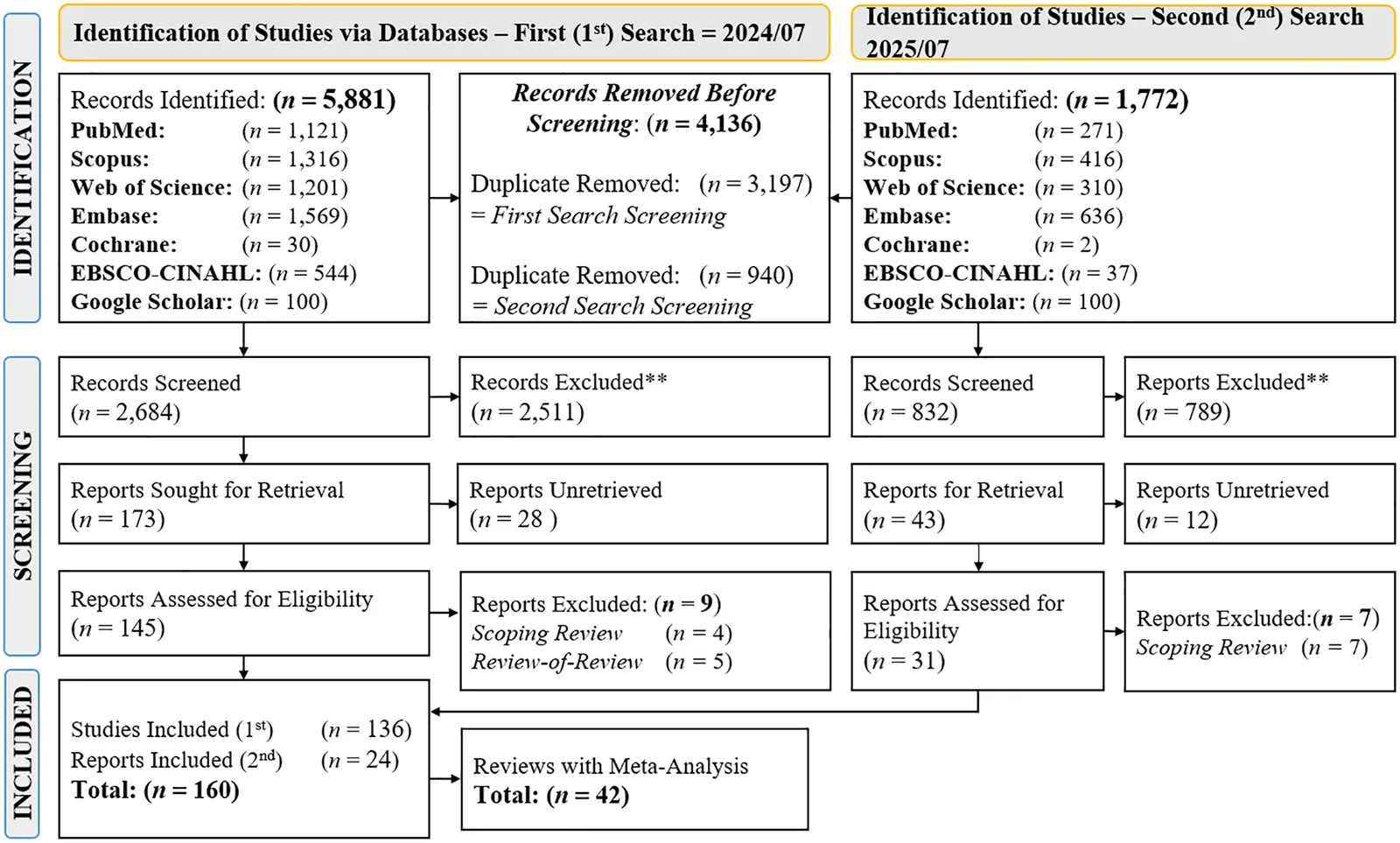
Flowchart of screening and inclusion of studies according to PRISMA 2020 guidelines [17].

The included reviews represented a wide range of clinical populations such as mental health conditions (n = 6; 13%), cardiovascular diseases (n = 8; 18%), diabetes or metabolic conditions (n = 8; 18%), cancer (n = 4), and surgical or rheumatic cases (n = 4). Smaller subsets addressed asthma (*n =* 3), COPD (*n =* 2), neurological conditions (*n =* 2), and HIV (*n =* 1). The majority of reviews (79%) only included adult populations.

### Digital health interventions

Intervention modalities varied extensively. Telehealth interventions were reported in half of the reviews, including teleconsultations (*n =* 12), telerehabilitation (*n =* 2), and telemonitoring (*n =* 7). mHealth interventions appeared in 62% of the reviews, featuring symptom trackers (*n =* 2), self-management applications (*n =* 23), and one lifestyle-focused app. Messaging technologies were reported in 60% of reviews, including SMS-based interventions (*n =* 17), interactive voice technologies (*n =* 5), and mobile reminders (*n =* 2). eHealth interventions were widespread, with 86% of reviews including patient portals (*n =* 11), interactive platforms (*n =* 19), or online education tools (*n =* 6). Wearable and sensor-based devices were reported in 19% (*n* = 8) of reviews, while social media–based interventions appeared in 12% of reviews (n = 5). Most reviews synthesised multiple intervention types; only 8 (18.2%) focused on a single modality.

### Outcomes

Treatment adherence was the most frequently reported primary PE outcome, with medication adherence evaluated in 15 reviews (36%) and non-medication adherence in 2 reviews (4.6%). Self-management outcomes appeared in 7 reviews, and patient satisfaction in 6. The most common secondary outcomes were disease control and quality of life (12 and 13 reviews, respectively), followed by implementation outcomes (7 reviews).

Outcome-specific CCA values for medication adherence, quality of life, HbA1c and anxiety scores were 0%, 0.83%, 2.71% and 1.95%, respectively. In the presence of this slight overlap, included reviews were retained in the meta-analysis. Pooled analysis in Fig 2 demonstrated that DHIs produced a moderate and significant improvement in medication adherence (*SMD* = 0.65; 95% CI: 0.22, 1.08; *I²* = 60.7%; *k* = 4, (n; represents the number of primary studies included in meta-analysis, k; represents the number of reviews included in meta-analysis) *n*  = 40), though moderate heterogeneity was detected. Secondary outcomes demonstrated consistent though heterogeneous effects. Six reviews contributed pooled estimates for quality-of-life outcomes. Fig 3 shows an aggregated SMD of 0.26 (95% CI: 0.11, 0.41; *I²* = 76.4%; *k* = 6, *n* = 71), though heterogeneity was high. As for clinical markers, pooled analysis showed a significant reduction in HbA1c (MD = −0.45%; 95% CI: −0.56, −0.34; *I²* = 0%, *k* = 3, *n* = 48) (Fig 4). DHIs were also associated with reductions in anxiety (*SMD =* −0.29; 95% CI: −0.54, −0.04; *I²* = 83.3%; *k* = 3, *n* = 36) (Fig 5).

**Fig 2:**
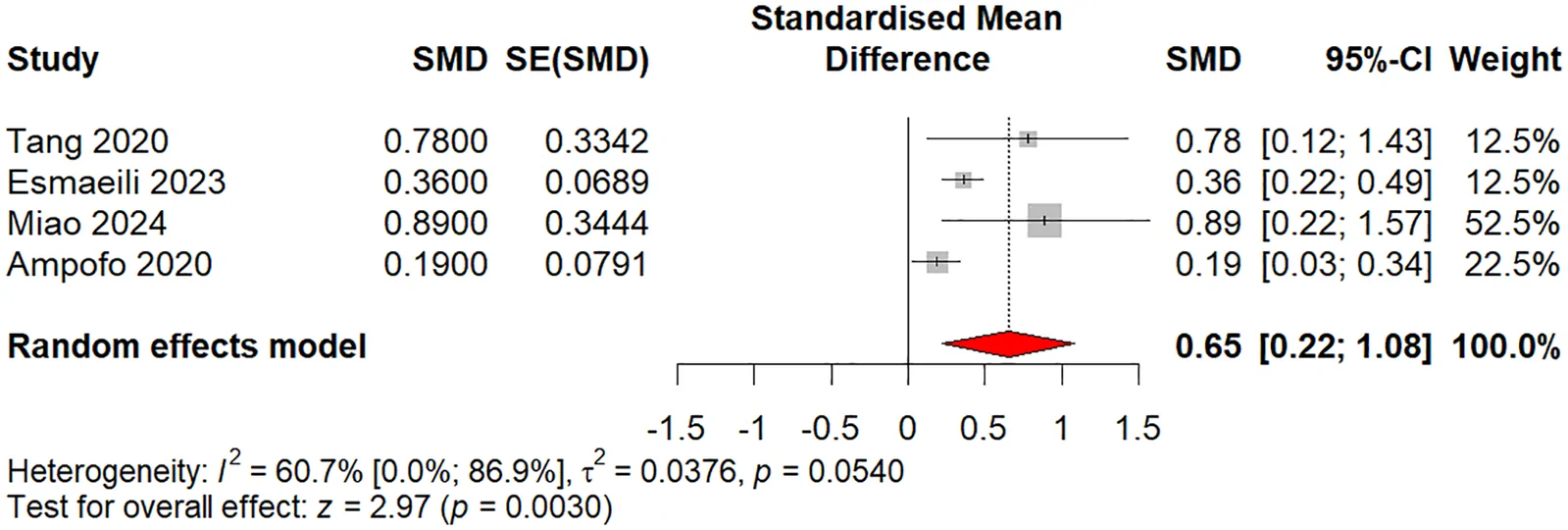
Forest plot showing standardised mean difference (SMD) of effect of DHIs on medication adherence. Larger effects are desirable, reflecting better medication adherence.

**Fig 3:**
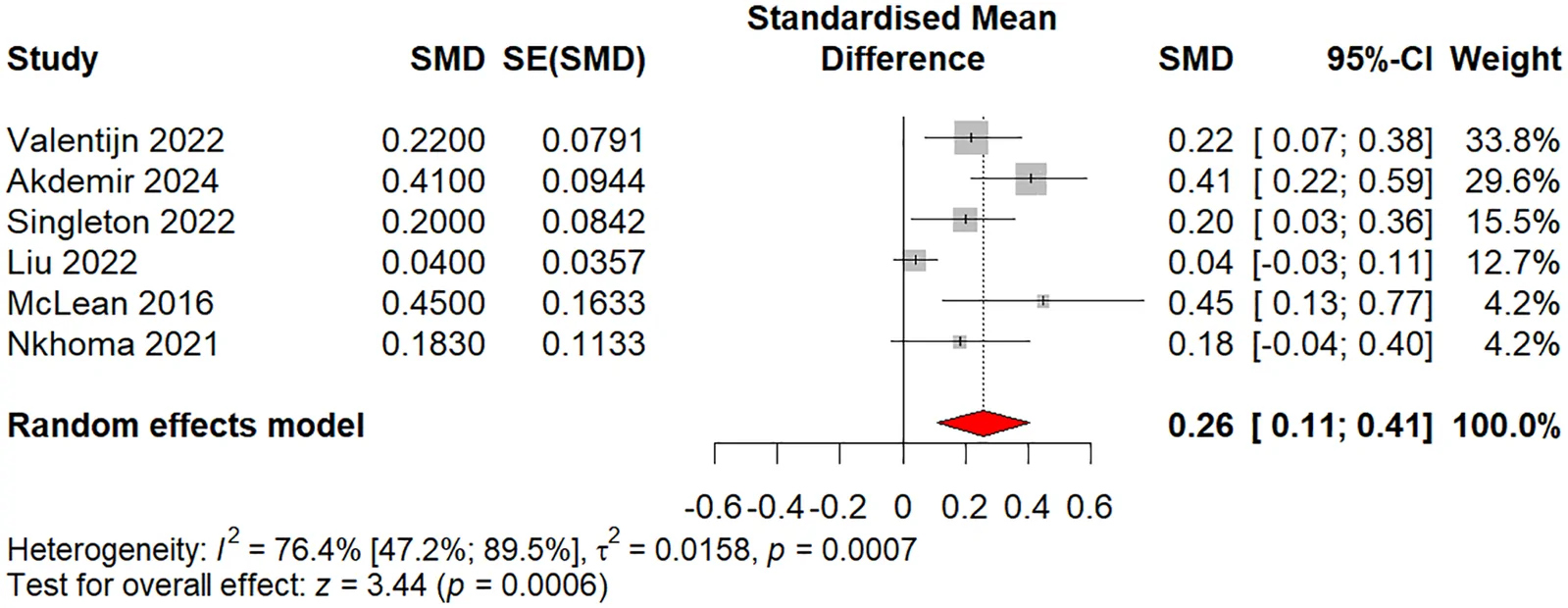
Pooled analysis showing standardised mean difference of DHIs on quality of life. Larger effects are desirable, indicating improved quality of life.

**Fig 4:**
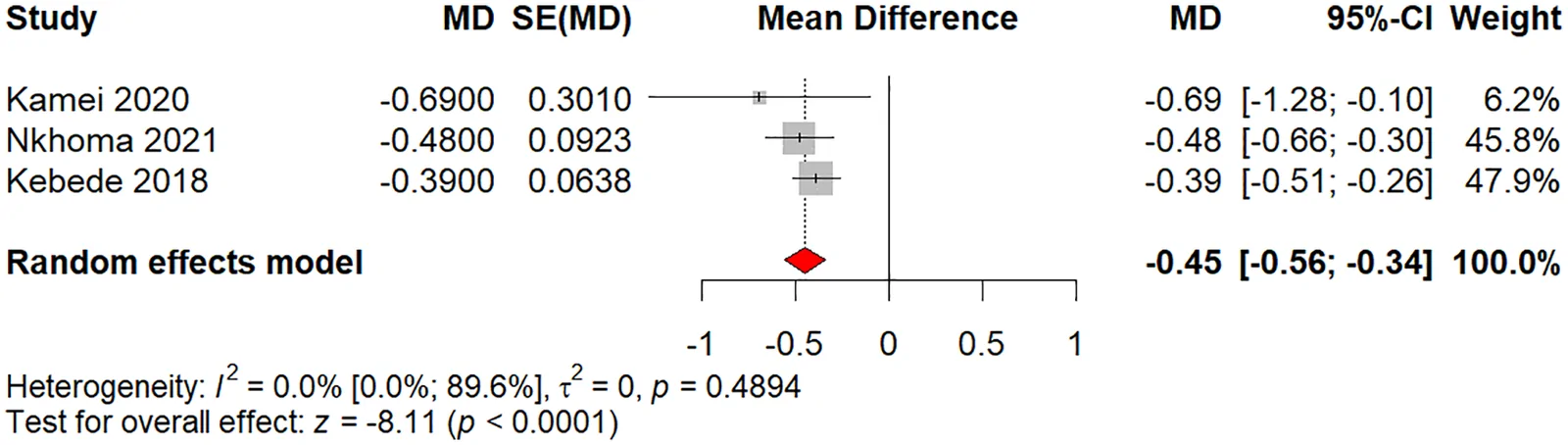
Pooled analysis showing the mean difference in the effect of DHIs on HbA1c. Larger negative effects are desirable, indicating reduced blood glucose concentrations. Pooled effect estimated from three reviews.

**Fig 5:**
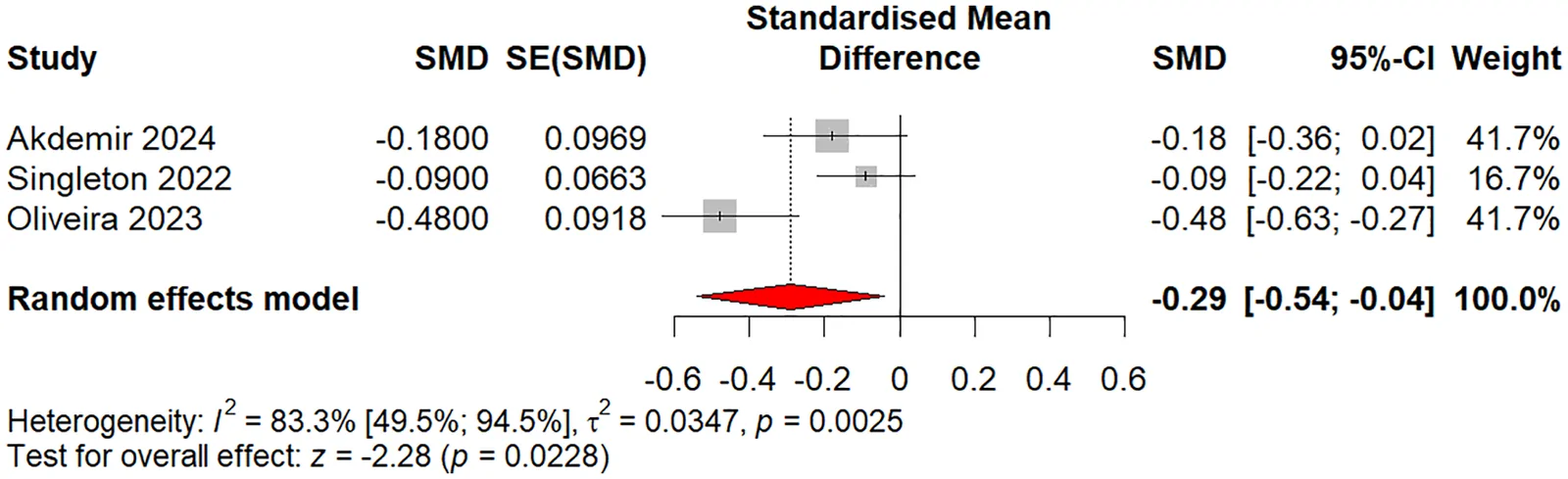
Pooled analysis shows standardised mean difference of DHIs on change in anxiety. Larger negative effects are desirable, indicating reduced anxiety scores. Pooled effect estimated from three reviews.

Due to small sample sizes and heterogeneity of reviews, key aspects of PE such as self-management, patient activation as well as patient outcomes such as asthma control scores, mental health markers such as depression, well-being and distress scores were not pooled statistically.

Subgroup analyses showed significant variation in effect estimates for quality-of-life and medication adherence by age group and condition (only for medication adherence) (Tables C-E in S1 Appendix File). However, low within-group sample sizes prevented subgroup analyses based on intervention type and AMSTAR-2 rating. Publication bias assessments were not conducted because all outcomes in the meta-analysis had fewer than 10 studies [68].

## Discussion

This umbrella review aimed to consolidate evidence on DHIs implemented across chronic disease populations to enhance patient engagement and health outcomes. Across 42 systematic reviews synthesising 853 primary studies and more than 574,676 participants, DHIs were evaluated in adults predominantly living with diabetes, cardiovascular diseases, mental health disorders, cancers, asthma, and COPD, conditions that account for the majority of global disability-adjusted life years (DALYs) according to WHO estimates [69]. These chronic conditions represent priority targets in the UN 2030 Sustainable Development Agenda [70], emphasising the importance of scalable interventions that strengthen PE as a behavioural and clinical determinant of health [1–3,6,7].

Most included reviews examined telehealth, eHealth, mHealth, and messaging interventions, reflecting the rapid digitalisation of healthcare catalysed by COVID-19 [8,9]. Evidence from RCTs, quasi-experimental designs, and observational studies contributed to the synthesis; however, 95% of the included systematic reviews were rated as low using AMSTAR-2. Despite available guidelines, methodological limitations such as lack of protocol registration and inadequate reporting of reasons for study exclusion persist, limiting transparency of study processes and confidence in review estimates, potentially leading to overestimation of evidence and its adoption [21]. Given this, findings should be interpreted as exploratory and as a call to improve the design and reporting of reviews in digital health and PE.

Despite these limitations, aggregated findings suggest that DHIs could improve key engagement outcomes, most notably medication adherence. Medication adherence showed a moderate pooled effect, although with some heterogeneity (*SMD* = 0.65; 95% CI: 0.22, 1.08), consistent with prior evidence [10,12,16]. DHIs also showed potential for selected secondary outcomes. Due to an insufficient number of contributing reviews, statistical pooling was not performed for blood pressure and disease control in asthma nor for other key primary outcomes, such as patient activation and self-management. A pooled reduction was, however, observed for HbA1c (MD = –0.45%; 95% CI: –0.56, –0.34). These findings align with prior evidence demonstrating positive DHI effects on glycaemic control [71–73].

Analysis of quality-of-life (QoL) suggested that small increases were likely (*SMD =* 0.26; MD = 0.08), in line with prior evidence that multi-component DHIs can improve HRQoL across chronic diseases [74–76]. QoL is multidimensional and often resistant to short-term change, especially among individuals with chronic multimorbidity [77,78]. Given that most primary studies were of short duration (<12 months), modest improvements in short-term studies would be expected. Subgroup analyses in this review found significant differences across age groups and conditions. Prior literature indicates that QoL and adherence may vary by age, condition severity, and comorbidity burden [78–80], necessitating a tailored approach in managing different patient populations. No subgroup analysis was undertaken to examine the individual effects of different DHI modalities due to an inadequate number of reviews within the subgroups. Nevertheless, different DHIs may be useful for different outcomes and steps across the engagement pathway.

### Strengths and limitations

Given the large number of systematic reviews in areas such as cardiovascular diseases and diabetes, this umbrella review offers a summary of the potential impact on both patient engagement and clinical outcomes to support evidence synthesis. The review has been conducted following a robust methodological approach guided by current umbrella review design guidelines [15,18].

However, the review also faces several limitations. First, most included reviews were of low quality. Furthermore, the review could not conduct subgroup analyses to single out findings from high-quality studies due to small sample sizes within subgroups. Despite higher-level synthesis, there was still an inadequate number of reviews to pool other critical aspects of patient engagement such as patient activation, satisfaction and health knowledge. Additionally, some pooled estimates were derived from a limited number of contributing reviews. With such limited data points, pooled effect sizes are statistically unstable and heterogeneity metrics (e.g., *I*²) are difficult to interpret meaningfully. Effects of gender or socio-economic status were not within the scope of the review. Additionally, included reviews are based on studies largely from developed nations, which may also limit generalisability to low- and middle-income countries (LMICs). Generalisability to communicable diseases also remains limited, despite their substantial burden in these LMICs.

### Future research

There is a need for further primary and secondary research in key areas related to the enhancement of patient activation, satisfaction and health knowledge through DHIs, areas that have been underrepresented so far, as well as how diverse patient and intervention characteristics, such as DHI modality and type, affect the overall impact of DHIs on both patient engagement and health outcomes. In terms of interventions, longer-term primary interventions are also required to assess the effectiveness of DHIs for patient engagement and more complex and time-sensitive patient outcomes. Equity considerations are required in future research on the topic as they were largely absent from the included reviews, especially for communicable diseases and populations living within low- and middle-income settings, who may also face the greatest barriers to digital access.

## Conclusion

This umbrella review synthesises a rapidly expanding but methodologically weak body of literature on digital health interventions for patient engagement. Evidence from 42 systematic reviews suggests that DHIs, across multiple digital modalities, may improve critical aspects of patient engagement such as medication adherence, especially for adult populations living with diabetes, cardiovascular and mental health disorders.

Study findings also suggest potential for DHIs to improve quality of life and selected clinical markers, highlighting the potential effects on both behavioural and health outcomes. However, lack of long-term investigations, compounded by the low methodological quality of existing reviews, limits confidence. Future research should prioritise robustly designed meta-analyses, explore underrepresented modalities such as social media, and examine differential effects across diverse populations. Until such evidence is available, policy and implementation decisions should proceed with caution.

## Supporting information

S1 Appendix Supplementary Material. Contains detailed outcome and intervention definitions (Table A), domain-level AMSTAR-2 quality assessments for each included review (Table B), subgroup analysis tables (Tables C–E), supplemental figures, and the full search strategies used for each database.
(DOCX)

S2 Appendix Literature Search and Extracted Data. Contains a numbered table of all excluded records with reasons for exclusion, a table of the 42 included reviews with data extractor names and eligibility confirmation, and a sheet of review-level extracted quantitative data used for the meta-analyses.
(XLSX)

S3 Appendix Analysis Code. R code and analysis inputs used to generate the pooled estimates and figures presented in this manuscript.
(R)

S1 PRISMA Checklist PRISMA Checklist. (DOCX)
