# Supplementary material for: Digital health interventions for patient engagement: A systematic umbrella review
Source: PLOS Digit Health. 2026 Oct 30;5(10):e0001785. doi: 10.1371/journal.pdig.0001785 (PMC13722202; doi:10.1371/journal.pdig.0001785)
Supplement: Supplementary file 1 [file pdig.0001785.s001.docx]

S1 APPENDIX FILE FOR DIGITAL HEALTH INTERVENTIONS FOR PATIENT ENGAGEMENT: A SYSTEMATIC UMBRELLA REVIEW

Contents

[Search Strategies per Database	7](#_Toc243467912)

[References	14](#_Toc243467913)

[List of Legends	17](#_Toc243467914)

Table A: Definition of interventions and outcomes

| **Category** | **Official Operational Definition** | **Source** |
| --- | --- | --- |
| **Telehealth / Telemedicine** | “The delivery of health care services, where distance is a critical factor, by all health care professionals using information and communication technologies.” | WHO Global Observatory for eHealth, *Telemedicine: Opportunities and Developments[43]* |
| **mHealth (Mobile Health)** | “Medical and public health practice supported by mobile devices, such as mobile phones, patient monitoring devices, personal digital assistants (PDAs), and other wireless devices.” | WHO mHealth Technical Evidence Review Group[44] |
| **Messaging (Targeted Client Communication)** | “Transmission of health-related content to individuals or groups through SMS, voice, email, or other digital channels for the purposes of improving health.” | WHO Digital Health Intervention Classification v1.0, 2018[45] |
| **eHealth** | “The use of information and communication technologies (ICT) for health.” | 2005 WHO eHealth Resolution WHA58.28[46] |
| **Wearables / Sensors (Personal Health Monitoring Devices)** | “Digital devices that collect health-related or biometric data from individuals in real time or near real time.” | OECD, *Using Digital Technologies to Improve the Recovery and Resilience of Health Systems[47]* |
| **Social Media** | “Platforms that enable user-generated content creation and social interaction, used by public health authorities for communication, community building, and behavioral influence.” | WHO, *Public Health and Social Measuresadvances in social listening for public health [48]* |
| **Gamification / Digital Games for Health** | “Digital games and game-based tools used to promote health behavior change or support clinical objectives.” | Bashar Alzghoul[49] |
| **Behavioral Engagement** | “Observable actions taken by people to maintain or improve their health, including adherence to treatment, lifestyle practices, and self-care behaviors.” | WHO Framework for Self-Care [50] |
| **Affective Engagement** | “Emotional responses and feelings that influence a person’s involvement in health care, including motivation, confidence, distress, or satisfaction.” | WHO People-Centred Care Framework[51] |
| **Cognitive Engagement** | “The knowledge, understanding, and information processing that individuals use in managing their own health.” | Measuring Cognitive Engagement [52] |
| **Patient Health Outcomes** | “Changes in health status attributable to health care, including symptoms, functional status, and quality of life.” | OECD Health Care Quality Indicators Framework [53] |
| **Health System Outcomes** | “Measures of how health systems perform, including efficiency, access, acceptability, safety, and financial protection.” | WHO Health Systems Performance Assessment[54] |

Table B: Quality Assessment done using AMSTAR-2 tool[55]

| **Author, Year, Country** | **1** | **2** | **3** | **4** | **5** | **6** | **7** | **8** | **9** | **10** | **11** | **12** | **13** | **14** | **15** | **16** | **Overall** |
| --- | --- | --- | --- | --- | --- | --- | --- | --- | --- | --- | --- | --- | --- | --- | --- | --- | --- |
| Abdelraheem 2024[1], Egypt | Y | N | Y | Y | Y | Y | N | Y | PY | N | Y | PY | PY | Y | N | Y | Low |
| Ampofo 2020[2], Australia | Y | N | Y | Y | Y | Y | N | Y | PY | N | Y | PY | PY | Y | N | Y | Low |
| Barakat 2019[3], Australia | Y | N | Y | Y | Y | Y | N | Y | PY | N | Y | PY | PY | Y | N | Y | Low |
| Valentijn 2022[4], Netherlands | Y | N | Y | Y | Y | Y | N | Y | PY | N | Y | PY | PY | Y | N | Y | Low |
| Verweel 2023[5], Canada | Y | N | Y | Y | Y | Y | N | Y | PY | N | Y | PY | PY | Y | N | Y | Low |
| Akdemir 2024[6], Australia | Y | N | Y | Y | Y | Y | N | Y | PY | N | Y | PY | PY | Y | N | Y | Low |
| Kamei 2020[7], Japan | Y | N | Y | Y | Y | Y | N | Y | PY | N | Y | PY | PY | Y | N | Y | Low |
| Labarca 2021[8], USA | Y | N | Y | Y | Y | Y | N | Y | PY | N | Y | PY | PY | Y | N | Y | Low |
| Butler 2022[9], Australia | Y | N | Y | Y | Y | Y | N | Y | PY | N | Y | PY | PY | Y | N | Y | Low |
| Miao 2024[10], China | Y | N | Y | Y | Y | Y | N | Y | PY | N | Y | PY | PY | Y | N | Y | Low |
| Singleton 2022[11], Australia | Y | N | Y | Y | Y | Y | N | Y | PY | N | Y | PY | PY | Y | N | Y | Low |
| Arsenijevic 2020[12], Netherlands | Y | N | Y | Y | Y | Y | N | Y | PY | N | Y | PY | PY | Y | N | Y | Low |
| Mcleanb 2016[13], UK | Y | N | Y | Y | Y | Y | N | Y | PY | N | Y | PY | PY | Y | N | Y | Low |
| Groot 2023[14], UK | Y | N | Y | Y | Y | Y | N | Y | PY | N | Y | PY | PY | Y | N | Y | Low |
| Doumen 2022[15], Belgium | Y | N | Y | Y | Y | Y | N | Y | PY | N | Y | PY | PY | Y | N | Y | Low |
| Domhardt 2021[16], Germany | Y | N | Y | Y | Y | Y | N | Y | PY | N | Y | PY | PY | Y | N | Y | Low |
| Parsons 2021[17], UK | Y | N | Y | Y | Y | Y | N | Y | PY | N | Y | PY | PY | Y | N | Y | Low |
| Liu 2022[18], China | Y | N | Y | Y | Y | Y | N | Y | PY | N | Y | PY | PY | Y | N | Y | Low |
| Nkhoma 2021[19], Taiwan | Y | N | Y | Y | Y | Y | N | Y | PY | N | Y | PY | PY | Y | N | Y | Low |
| Bunting 2021[20], UK | Y | N | Y | Y | Y | Y | N | Y | PY | N | Y | PY | PY | Y | N | Y | Low |
| So 2018[21], Hong Kong | Y | N | Y | Y | Y | Y | N | Y | PY | N | Y | PY | PY | Y | N | Y | Low |
| Esmaeili 2023[22], Iran | Y | N | Y | Y | Y | Y | N | Y | PY | N | Y | PY | PY | Y | N | Y | Low |
| Bevens 2022[23], Australia | Y | N | Y | Y | Y | Y | N | Y | PY | N | Y | PY | PY | Y | N | Y | Low |
| Alkhaldi 2016[24], UK | Y | N | Y | Y | Y | Y | N | Y | PY | N | N/A | PY | PY | N/A | N | Y | Critically Low |
| Sin 2020[25], UK | Y | N | Y | Y | Y | Y | N | Y | PY | N | Y | PY | PY | Y | N | Y | Low |
| Aardoom 2020[26], Netherlands | Y | N | Y | Y | Y | Y | N | Y | PY | N | Y | PY | PY | Y | N | Y | Low |
| McLean 2015[27], UK | Y | N | Y | Y | Y | Y | N | Y | PY | N | Y | PY | PY | Y | N | Y | Low |
| Pauley 2023[28], Netherlands | Y | N | Y | Y | Y | Y | N | Y | PY | N | Y | PY | PY | Y | N | Y | Low |
| Jeminiwa 2019[29], USA | Y | N | Y | Y | Y | Y | N | Y | PY | N | Y | PY | PY | Y | N | Y | Low |
| Tang 2020[30], Australia | Y | N | Y | Y | Y | Y | N | Y | PY | N | Y | PY | PY | Y | N | Y | Low |
| Vitija 2022[31], UK | Y | N | Y | Y | Y | Y | N | Y | PY | N | Y | PY | PY | Y | N | Y | Low |
| Oliveira 2023[32], Portugal | Y | N | Y | Y | Y | Y | N | Y | P^[1]^Y | N | Y | PY | PY | Y | N | Y | Low |
| Chan 2022[33], New Zealand | Y | N | Y | Y | Y | Y | N | Y | PY | N | Y | PY | PY | Y | N | Y | Low |
| Kebede 2018[34], Germany | Y | N | Y | Y | Y | Y | N | Y | PY | N | Y | PY | PY | Y | N | Y | Low |
| Seiler 2017[35], USA | Y | N | Y | Y | Y | Y | N | Y | PY | N | Y | PY | PY | Y | N | Y | Low |
| Khoong 2021[36], USA | Y | N | Y | Y | Y | Y | N | Y | PY | N | Y | PY | PY | Y | N | Y | Low |
| Goldberg 2025[37], Germany | Y | N | Y | Y | Y | Y | N | Y | PY | N | Y | PY | PY | Y | N | Y | Low |
| Osovskaya 2025[38], UK | Y | N | Y | Y | Y | Y | N | Y | PY | N | N/A | PY | PY | N/A | N | Y | Critically Low |
| Zhuang 2025[39], Malaysia | Y | N | Y | Y | Y | Y | N | Y | PY | N | Y | PY | PY | Y | N | Y | Low |
| Ansari 2025[40], UK | Y | N | Y | Y | Y | Y | N | Y | PY | N | Y | PY | PY | Y | N | Y | Low |
| Cevasco 2024[41], USA | Y | N | Y | Y | Y | Y | N | Y | PY | N | Y | PY | PY | Y | N | Y | Low |
| Getie 2025[42], Ethiopia | Y | N | Y | Y | Y | Y | N | Y | PY | N | Y | PY | PY | Y | N | Y | Low |

Table C: Random-Effects Subgroup Analysis by Participant Age for Quality of Life

| **Participants** | **k** | **SMD (Pooled)** | **95% CI** | ***I² (%)*** |
| --- | --- | --- | --- | --- |
| Adults | 7 | 0.075 | [0.04, 0.11] | 67.40 |
| Mixed Ages | 3 | 0.267 | [0.12, 0.41] | 42.26 |

*Note. k = number of included studies contributing to the pooled effect size estimate within each subgroup. Test of subgroup differences: Q₍₂₎ = 21.87, p = 0.000.*

Table D: Random-Effects Subgroup Analysis by Participant Condition for Quality of Life

| **Condition Category** | **k** | **SMD (Pooled)** | **95% CI** | ***I² (%)*** |
| --- | --- | --- | --- | --- |
| Cancers | 2 | 0.301 | [0.10, 0.51] | 63.73 |
| Cardio-metabolic | 2 | 0.070 | [-0.04, 0.18] | 29.32 |
| Others | 2 | 0.291 | [0.08, 0.50] | 37.79 |

*Note. k = number of included studies contributing to the pooled effect size estimate within each subgroup. Test of subgroup differences: Q₍₂₎ = 5.78, p = 0.056.*

Table E: Random-Effects Subgroup Analysis by Participant Condition for Medication Adherence

| **Condition Category** | **k** | **SMD (Pooled)** | **95% CI** | ***I² (%)*** |
| --- | --- | --- | --- | --- |
| Cardio-metabolic | 2 | 0.460 | [-0.21, 1.13] | 74.52 |
| Others | 2 | 0.443 | [0.12, 0.77] | 34.0 |

*Note. k = number of included studies contributing to the pooled effect size estimate within each subgroup. Test of subgroup differences: Q₍₂₎ = 5.44, p = 0.020.*

# Search Strategies per Database

**Data Search Terms – Conducted on: Data Search Terms-20240709**

Search string for databases for Scopus, PubMed, Web of Science (WOS), Cochrane, Embase/EBSCO-CINAHL, the combined keywords and sub-keywords using Boolean operators (AND, OR) and parentheses for grouping.

**Define Search Result- RIS & CSV File**

**PubMed**

(("digital intervention*" OR "eHealth" OR "mHealth" OR "mobile health" OR "telehealth" OR "telemedicine" OR "e-Health" OR "digital health"OR "internet intervention*" OR "online intervention*") AND ("patient engagement" OR "patient participation" OR "patient involvement" OR "Patient Activation" OR "patient empowerment" OR "self-management" OR "adherence" OR "patient satisfaction") AND ("systematic review" OR "meta-analysis"))

**Define: 20240709: 1,121 Found - 3:14:41**

Search: (("digital intervention*" OR "eHealth" OR "mHealth" OR "mobile health" OR "telehealth" OR "telemedicine" OR "e-Health" OR "digital health"OR "internet intervention*" OR "online intervention*") AND ("patient engagement" OR "patient participation" OR "patient involvement" OR "Patient Activation" OR "patient empowerment" OR "self-management" OR "adherence" OR "patient satisfaction") AND ("systematic review" OR "meta-analysis")) Filters: Full text, Meta-Analysis, Systematic Review, English

**Define: 20250706: 271 Found - 5:57:40**

**Scopus**

( ( "digital intervention*" OR "eHealth" OR "mHealth" OR "mobile health" OR "telehealth" OR "telemedicine" OR "e-Health" OR "digital health" OR "internet intervention*" OR "online intervention*" ) AND ( "patient engagement" OR "patient participation" OR "patient involvement" OR "Patient Activation" OR "patient empowerment" OR "self-management" OR "adherence" OR "patient satisfaction" ) AND ( "systematic review" OR "meta-analysis" ) )

**Define: 20240709: 1,316 Found - 3:25:38**

TITLE-ABS-KEY((("digital intervention*" OR "eHealth" OR "mHealth" OR "mobile health" OR "telehealth" OR "telemedicine" OR "e-Health" OR "digital health"OR "internet intervention*" OR "online intervention*") AND ("patient engagement" OR "patient participation" OR "patient involvement" OR "Patient Activation" OR "patient empowerment" OR "self-management" OR "adherence" OR "patient satisfaction") AND ("systematic review" OR "meta-analysis"))) AND ( LIMIT-TO ( LANGUAGE,"English" ) ) AND ( LIMIT-TO ( EXACTKEYWORD,"Systematic Review" ) OR LIMIT-TO ( EXACTKEYWORD,"Meta Analysis" ) )

**Define: 20250706: 416 Found - 6:10:50**

**Web of Science (WOS)**

( ( "digital intervention*" OR "eHealth" OR "mHealth" OR "mobile health" OR "telehealth" OR "telemedicine" OR "e-Health" OR "digital health" OR "internet intervention*" OR "online intervention*" ) AND ( "patient engagement" OR "patient participation" OR "patient involvement" OR "Patient Activation" OR "patient empowerment" OR "self-management" OR "adherence" OR "patient satisfaction" ) AND ( "systematic review" OR "meta-analysis" ) )

**Define: 20240709: 1,201 Found - 4:01:38**

( ( "digital intervention*" OR "eHealth" OR "mHealth" OR "mobile health" OR "telehealth" OR "telemedicine" OR "e-Health" OR "digital health" OR "internet intervention*" OR "online intervention*" ) AND ( "patient engagement" OR "patient participation" OR "patient involvement" OR "Patient Activation" OR "patient empowerment" OR "self-management" OR "adherence" OR "patient satisfaction" ) AND ( "systematic review" OR "meta-analysis" ) ) (All Fields) and English (Languages)

**Define: 20250706: 310 Found - 6:12:05**

**Cochrane Library**

("digital NEXT intervention" OR "eHealth" OR "mHealth" OR "mobile health" OR "telehealth" OR "telemedicine" OR "e-Health" OR "digital health" OR "internet NEXT intervention" OR "online NEXT intervention") AND ("patient engagement" OR "patient participation" OR "patient involvement" OR "Patient Activation" OR "patient empowerment" OR "self-management" OR "adherence" OR "patient satisfaction")

**Define: 20240709: 30 Found - 4:48:48**

30 Cochrane Reviews matching ("digital NEXT intervention" OR "eHealth" OR "mHealth" OR "mobile health" OR "telehealth" OR "telemedicine" OR "e-Health" OR "digital health" OR "internet NEXT intervention" OR "online NEXT intervention") AND ("patient engagement" OR "patient participation" OR "patient involvement" OR "Patient Activation" OR "patient empowerment" OR "self-management" OR "adherence" OR "patient satisfaction") in Title Abstract Keyword

**Define: 20250706: 2 Found - 7:25:30**

**EMBASE**

( "digital intervention*" OR "eHealth" OR "mHealth" OR "mobile health" OR "telehealth" OR "telemedicine" OR "e-Health" OR "digital health" OR "internet intervention*" OR "online intervention*" ) AND ( "patient engagement" OR "patient participation" OR "patient involvement" OR "Patient Activation" OR "patient empowerment" OR "self-management" OR "adherence" OR "patient satisfaction" ) AND ( "systematic review" OR "meta-analysis" )

**Define: 20240709: 1,569 Found - 4:18:48**

('digital intervention*' OR 'ehealth' OR 'mhealth' OR 'mobile health' OR 'telehealth' OR 'telemedicine' OR 'e-health' OR 'digital health' OR 'internet intervention*' OR 'online intervention*') AND ('patient engagement' OR 'patient participation' OR 'patient involvement' OR 'Patient Activation' OR 'patient empowerment' OR 'self-management' OR 'adherence' OR 'patient satisfaction') AND ('systematic review' OR 'meta-analysis')

**Define: 20250706: 636 Found - 6:51:15**

**EBSCOHost- CINAHL Plus**

( "digital intervention*" OR "eHealth" OR "mHealth" OR "mobile health" OR "telehealth" OR "telemedicine" OR "e-Health" OR "digital health" OR "internet intervention*" OR "online intervention*" ) AND ( "patient engagement" OR "patient participation" OR "patient involvement" OR "Patient Activation" OR "patient empowerment" OR "self-management" OR "adherence" OR "patient satisfaction" ) AND ( "systematic review" OR "meta-analysis" )

**Define: 20240709: 544 Found - 5:24:16**

('digital intervention*' OR 'ehealth' OR 'mhealth' OR 'mobile health' OR 'telehealth' OR 'telemedicine' OR 'e-health' OR 'digital health' OR 'internet intervention*' OR 'online intervention*') AND ('patient engagement' OR 'patient participation' OR 'patient involvement' OR 'Patient Activation' OR 'patient empowerment' OR 'self-management' OR 'adherence' OR 'patient satisfaction') AND ('systematic review' OR 'meta-analysis')

('digital intervention*' OR 'ehealth' OR 'mhealth' OR 'mobile health' OR 'telehealth' OR 'telemedicine' OR 'e-health' OR 'digital health' OR 'internet intervention*' OR 'online intervention*') AND ('patient engagement' OR 'patient participation' OR 'patient involvement' OR 'Patient Activation' OR 'patient empowerment' OR 'self-management' OR 'adherence' OR 'patient satisfaction') AND ('systematic review' OR meta analysis OR meta-analysis)

**Define: 20250708: 37 Found -6:43:16**

# References

1.	Abdelraheem, O., M. Salama, and S. Chun, *Impact of digital interventions and online health communities in patient activation: Systematic review and meta-analysis.* International Journal of Medical Informatics, 2024. **188**: p. 105481.

2.	Ampofo, A.G., E. Khan, and M.B. Ibitoye, *Understanding the role of educational interventions on medication adherence in hypertension: A systematic review and meta-analysis.* Heart &amp; Lung, 2020. **49**(5): p. 537–547.

3.	Barakat, S., et al., *Evaluating the role of digital intervention design in treatment outcomes and adherence to eTherapy programs for eating disorders: A systematic review and meta‐analysis.* International Journal of Eating Disorders, 2019. **52**(10): p. 1077–1094.

4.	Valentijn, P.P., et al., *Digital Health Interventions for Musculoskeletal Pain Conditions: Systematic Review and Meta-analysis of Randomized Controlled Trials.* Journal of Medical Internet Research, 2022. **24**(9): p. e37869.

5.	Verweel, L., et al., *The effect of digital interventions on related health literacy and skills for individuals living with chronic diseases: A systematic review and meta-analysis.* International Journal of Medical Informatics, 2023. **177**: p. 105114.

6.	Akdemir, A., et al., *Guided versus non‐guided digital psychological interventions for cancer patients: A systematic review and meta‐analysis of engagement and efficacy.* Psycho-Oncology, 2024. **33**(1).

7.	Kamei, T., et al., *The use of wearable devices in chronic disease management to enhance adherence and improve telehealth outcomes: A systematic review and meta-analysis.* Journal of Telemedicine and Telecare, 2022. **28**(5): p. 342–359.

8.	Labarca, G., et al., *Telemedicine interventions for CPAP adherence in obstructive sleep apnea patients: Systematic review and meta-analysis.* Sleep Medicine Reviews, 2021. **60**: p. 101543.

9.	Butler, S., et al., *Effectiveness of eHealth and mHealth Interventions Supporting Children and Young People Living With Juvenile Idiopathic Arthritis: Systematic Review and Meta-analysis.* Journal of Medical Internet Research, 2022. **24**(2): p. e30457.

10.	Miao, Y., et al., *Effectiveness of eHealth Interventions in Improving Medication Adherence Among Patients With Cardiovascular Disease: Systematic Review and Meta-Analysis.* Journal of Medical Internet Research, 2024. **26**: p. e58013.

11.	Singleton, A.C., et al., *Electronic Health Interventions for Patients With Breast Cancer: Systematic Review and Meta-Analyses.* Journal of Clinical Oncology, 2022. **40**(20): p. 2257–2270.

12.	Arsenijevic, J., L. Tummers, and N. Bosma, *Adherence to Electronic Health Tools Among Vulnerable Groups: Systematic Literature Review and Meta-Analysis.* Journal of Medical Internet Research, 2020. **22**(2): p. e11613.

13.	McLean, G., et al., *Interactive digital interventions to promote self-management in adults with asthma: systematic review and meta-analysis.* BMC Pulmonary Medicine, 2016. **16**(1).

14.	Groot, J., et al., *The Effectiveness of Fully Automated Digital Interventions in Promoting Mental Well-Being in the General Population: Systematic Review and Meta-Analysis.* JMIR Mental Health, 2023. **10**: p. e44658.

15.	Doumen, M., et al., *Engagement and attrition with eHealth tools for remote monitoring in chronic arthritis: a systematic review and meta-analysis.* RMD Open, 2022. **8**(2): p. e002625.

16.	Domhardt, M., et al., *Therapeutic processes in digital interventions for anxiety: A systematic review and meta-analytic structural equation modeling of randomized controlled trials.* Clinical Psychology Review, 2021. **90**: p. 102084.

17.	Parsons, J., et al., *How effective are digital interventions in increasing flu vaccination among pregnant women? A systematic review and meta-analysis.* Journal of Public Health, 2022. **44**(4): p. 863–876.

18.	Liu, S., et al., *Effectiveness of eHealth Self-management Interventions in Patients With Heart Failure: Systematic Review and Meta-analysis.* Journal of Medical Internet Research, 2022. **24**(9): p. e38697.

19.	Nkhoma, D.E., et al., *Digital interventions self-management education for type 1 and 2 diabetes: A systematic review and meta-analysis.* Computer Methods and Programs in Biomedicine, 2021. **210**: p. 106370.

20.	Bunting, J.W., et al., *Digital interventions for promoting exercise adherence in chronic musculoskeletal pain: a systematic review and meta-analysis.* Physiotherapy, 2021. **111**: p. 23–30.

21.	So, C.F. and J.W. Chung, *Telehealth for diabetes self-management in primary healthcare: A systematic review and meta-analysis.* Journal of Telemedicine and Telecare, 2018. **24**(5): p. 356–364.

22.	Esmaeili, E.D., et al., *Does telehealth affect the adherence to ART among patients with HIV? A systematic review and meta-analysis.* BMC Infectious Diseases, 2023. **23**(1).

23.	Bevens, W., et al., *Attrition Within Digital Health Interventions for People With Multiple Sclerosis: Systematic Review and Meta-analysis.* Journal of Medical Internet Research, 2022. **24**(2): p. e27735.

24.	Alkhaldi, G., et al., *The Effectiveness of Prompts to Promote Engagement With Digital Interventions: A Systematic Review.* Journal of Medical Internet Research, 2016. **18**(1): p. e6.

25.	Sin, J., et al., *Digital Interventions for Screening and Treating Common Mental Disorders or Symptoms of Common Mental Illness in Adults: Systematic Review and Meta-analysis.* Journal of Medical Internet Research, 2020. **22**(9): p. e20581.

26.	Aardoom, J.J., et al., *Effectiveness of Electronic Health Interventions in Improving Treatment Adherence for Adults With Obstructive Sleep Apnea: Meta-Analytic Review.* Journal of Medical Internet Research, 2020. **22**(2): p. e16972.

27.	McLean, G., et al., *Digital interventions to promote self-management in adults with hypertension systematic review and meta-analysis.* Journal of Hypertension, 2016. **34**(4): p. 600–612.

28.	Pauley, D., et al., *Two decades of digital interventions for anxiety disorders: a systematic review and meta-analysis of treatment effectiveness.* Psychological Medicine, 2021: p. 1–13.

29.	Jeminiwa, R., et al., *Impact of eHealth on medication adherence among patients with asthma: A systematic review and meta-analysis.* Respiratory Medicine, 2019. **149**: p. 59–68.

30.	Tang, J., et al., *eHealth Interventions for Solid Organ Transplant Recipients: A Systematic Review and Meta-analysis of Randomized Controlled Trials.* Transplantation, 2020. **104**(8): p. e224–e235.

31.	Vitija, A., A. Amirthalingam, and A. Soltani, *The impact of digital interventions on medication adherence in paediatric populations with attention deficit hyperactivity disorder, depression, and/or anxiety: A rapid systematic review and meta-analysis.* Research in Social and Administrative Pharmacy, 2022. **18**(12): p. 4017–4027.

32.	Oliveira, C., et al., *Internet-delivered cognitive behavioral therapy for anxiety among university students: A systematic review and meta-analysis.* Internet Interventions, 2023. **31**: p. 100609.

33.	Chan, A., et al., *Digital interventions to improve adherence to maintenance medication in asthma.* Cochrane Database of Systematic Reviews, 2022. **2022**(6).

34.	Kebede, M.M., et al., *Effectiveness of Digital Interventions for Improving Glycemic Control in Persons with Poorly Controlled Type 2 Diabetes: A Systematic Review, Meta-analysis, and Meta-regression Analysis.* Diabetes Technology &amp; Therapeutics, 2018. **20**(11): p. 767–782.

35.	Seiler, A., et al., *eHealth and mHealth interventions in the treatment of fatigued cancer survivors: A systematic review and meta-analysis.* Psycho-Oncology, 2017. **26**(9): p. 1239–1253.

36.	Khoong, E.C., et al., *Mobile health strategies for blood pressure self-management in urban populations with digital barriers: systematic review and meta-analyses.* npj Digital Medicine, 2021. **4**(1).

37.	Goldberg, N., et al., *Sociodemographic and Socioeconomic Determinants for the Usage of Digital Patient Portals in Hospitals: Systematic Review and Meta-Analysis on the Digital Divide.* Journal of Medical Internet Research, 2025. **27**: p. e68091.

38.	Osovskaya, I., A. Blandford, and H.W. Potts, *A systematic review of the effect of personal health records on patient activation.* DIGITAL HEALTH, 2025. **11**.

39.	Zhuang, M., et al., *Effectiveness of Digital Health Interventions for Chronic Obstructive Pulmonary Disease: Systematic Review and Meta-Analysis.* Journal of Medical Internet Research, 2025. **27**: p. e76323.

40.	Ansari, S., et al., *Comparing the Outcomes of Digital and Traditional Cardiac Rehabilitation Practices: A Systematic Review and Meta-Analysis.* Cureus, 2025.

41.	Cevasco, K.E., et al., *Patient Engagement with Conversational Agents in Health Applications 2016–2022: A Systematic Review and Meta-Analysis.* Journal of Medical Systems, 2024. **48**(1).

42.	Getie, A., et al., *Assessing the impact of telehealth on blood glucose management among patients with diabetes: a systematic review and meta-analysis of randomized controlled trials.* BMC Health Services Research, 2025. **25**(1).

43.	World Health Organization, *Telemedicine: opportunities and developments in Member States: report on the second global survey on eHealth*

*2009.*, in *Global Observatory for eHealth Series*. 2010, World Health Organization: Geneva.

44.	Agarwal, S., et al., *Guidelines for reporting of health interventions using mobile phones: mobile health (mHealth) evidence reporting and assessment (mERA) checklist.* BMJ, 2016: p. i1174.

45.	World Health Organization, *Classification of Digital Health Interventions v1.0*. 2018.

46.	World Health Organization, *WHA58.28 eHealth*. 2005, World Health Assembly: Geneva.

47.	Organisation for Economic Co-operation and Development, *Digitalisation of health systems can significantly improve performance and outcomes*, in *Newsroom*, OECD, Editor. 2023, Organisation for Economic Co-operation and Development,.

48.	World Health Organization, *WHO fourth global infodemic management conference: advances in social listening for public health*. 2023: Geneva.

49.	Alzghoul, B., *The Effectiveness of Gamification in Changing Health-related Behaviors: A Systematic Review and Meta-analysis.* The Open Public Health Journal, 2024. **17**(1).

50.	World Health Organization, *WHO guideline on self-care interventions for health and well-being, 2022 revision*. 2022, World Health Organization: Geneva.

51.	World Health Organization, *Framework on integrated, people centred health services*. 2015, World Health Organization,: Geneva.

52.	Li, S., *Measuring Cognitive Engagement: An Overview of Measurement Instruments and Techniques.* International Journal of Psychology and Educational Studies, 2022. **8**(3): p. 63–76.

53.	Edward Kelley, J.H., *Health Care Quality Indicators Project: Conceptual Framework Paper*, in *OECD Health Working Papers No. 23*. 2006, OECD

54.	World Health Organization, *Health system performance assessment: A framework for policy analysis*. 2022: Geneva.

55.	Shea, B.J., et al., *AMSTAR 2: a critical appraisal tool for systematic reviews that include randomised or non-randomised studies of healthcare interventions, or both.* BMJ, 2017. **358**: p. j4008.

# List of Legends

**Tables**

[Table A: Definition of interventions and outcomes	2](#_Toc243467919)

[Table B: Quality Assessment done using AMSTAR-2 tool[55]	4](#_Toc243467920)

[Table C: Random-Effects Subgroup Analysis by Participant Age for Quality of Life	6](#_Toc243467921)

[Table D: Random-Effects Subgroup Analysis by Participant Condition for Quality of Life	6](#_Toc243467922)

[Table E: Random-Effects Subgroup Analysis by Participant Condition for Medication Adherence	6](#_Toc243467923)

1. Y; Yes, N; No, PY; Partially Yes, N/A; Not Applicable
