# Supplementary material for: Digital health interventions for patient engagement: A systematic umbrella review
Source: PLOS Digit Health. 2026 Oct 30;5(10):e0001785. doi: 10.1371/journal.pdig.0001785 (PMC13722202; doi:10.1371/journal.pdig.0001785)
Supplement: Supplementary file 3 [file pdig.0001785.s006.docx]

**Response to Reviewers**

**A. Journal Requirements**

**Requirement 1:** Please provide separate figure files in .tif or .eps format.

**Response:** All manuscript figures have been provided as separate TIFF files, processed using PLOS's NAAS tool to confirm they meet print size and resolution requirements.

**Requirement 2:** Please provide an Author Summary. This should appear in your manuscript between the Abstract (if applicable) and the Introduction and should be 150–200 words long.

**Response:** An Author Summary has been added immediately below the main Abstract and before the Introduction (word count: 168).

**Requirement 3:** We have noticed that you have uploaded Supporting Information files, but you have not included a list of legends. Please add a full list of legends for your Supporting Information files after the references list.

**Response:** A full list of legends for the Supporting Information files (S1–S3) has been added after the reference list, describing the contents of each file.

**Requirement 4:** As required by our policy on Data Availability, please ensure your manuscript or supplementary information includes: (a) a numbered table of all studies identified in the literature search, including those excluded, with reasons for exclusion; (b) a table of all data extracted from the primary research sources, including extractor names, dates, eligibility confirmation, and all data needed to replicate the analyses; (c) if applicable, a table of completed risk of bias/quality assessments for each study or outcome, by domain; and an explanation of how missing data were handled.

**Response:** We have moved the table of all records assessed during the literature search, including excluded studies with reasons for exclusion, to the S2 File, sheet ‘Literature search’. All records identified at this stage were published reviews. All information extracted for the narrative synthesis (S2 File, sheet ‘Included Reviews (n=42)’) and for the statistical analysis (S2 File, sheet ‘Extracted quantitative data’) has been transferred from our project repository into the S2 File; all included reviews now have the name of the data extractor recorded. A completed domain-level AMSTAR-2 quality assessment for every included review is provided in the S1 File, Table 5, and this is referenced in the first paragraph of the Results section (‘Study Characteristics’). An explanation of how missing data were handled has been added to the Methods section (data extraction and synthesis paragraph): one article had missing information on overall sample size; as this article was not included in the meta-analysis, the missing data did not affect the statistical analysis.

**Requirement 5:** Please note that your Data Availability Statement is currently missing the repository name and the DOI/accession number of each dataset OR a direct link to access each database.

**Response:** The Data Availability Statement has been updated. In line with the recommendations above, all data previously held in an external repository have been transferred into the Supporting Information files (S1–S3); the manuscript is therefore no longer accompanied by an external repository, and the statement now reflects this directly.

**B. Additional Editor Comments**

**Editor:** The two existing reviews split sharply: one recommends Accept with minimal comment, the other flags the same AMSTAR-2/interpretation problem I'm raising here and recommends Major Revision. Given the size of that gap, and that the more critical review is also the more substantive one, I'd weight it heavily in the decision. The Accept review doesn't engage with the quality-of-evidence issue at all, which is the paper's central weakness. Worth flagging for production before the next round: the abstract truncation in the submitted PDF should be confirmed as a file error and not an actual incomplete abstract, since it affects how reviewers are reading the conclusions section.

**Response:** We appreciate the editor's decision to weight Reviewer #2's more substantive critique, and we have made the quality-of-evidence/interpretation issue the central organizing concern of this revision (see response to Reviewer #2, comment 1, below). We can confirm that the abstract truncation observed in the originally submitted PDF was a file conversion/production error and not a defect in the manuscript: the complete abstract (209 words) has been present in the manuscript throughout, and we have re-checked the PDF conversion for this resubmission to ensure it displays in full.

**C. Reviewer #1**

1. Does this manuscript meet PLOS Digital Health's publication criteria? Reviewer #1: Yes.

2. Has the statistical analysis been performed appropriately and rigorously? Reviewer #1: Yes.

3. Have the authors made all data underlying the findings fully available? Reviewer #1: Yes.

4. Is the manuscript presented in an intelligible fashion and written in standard English? Reviewer #1: Yes.

**Comments to the Author:** 1. After reviewing PLOS Digital Health's publication criteria, this paper does fulfill the criteria. 2. The statistical analysis has reasoning behind the incorporation of the analysis into the paper. 3. The data and supplemental material is available. 4. It is understandable.

**Response:** We thank Reviewer #1 for their positive assessment of the manuscript's methodological soundness, statistical analysis, data availability, and clarity. No specific changes were requested; however, the manuscript has been substantially revised in response to Reviewer #2's comments, and we believe these revisions further strengthen the aspects Reviewer #1 highlighted as satisfactory.

**D. Reviewer #2**

1. Does this manuscript meet PLOS Digital Health's publication criteria? Reviewer #2: Partly.

2. Has the statistical analysis been performed appropriately and rigorously? Reviewer #2: Yes.

3. Have the authors made all data underlying the findings fully available? Reviewer #2: Yes.

4. Is the manuscript presented in an intelligible fashion and written in standard English? Reviewer #2: Yes.

**Comment 1 (Primary concern):** The authors report that 40 of 42 included reviews (95%) were rated low or critically low-quality using AMSTAR-2. Despite this, the abstract and conclusions present pooled estimates as though they carry confirmatory weight. The authors acknowledge this limitation but never resolve the tension; the manuscript cannot simultaneously claim that the evidence is severely constrained while presenting effect sizes as reliable. The authors should either reframe the entire paper as a methodological cautionary tale emphasizing the poor quality of the evidence base, or substantially downgrade the prominence of pooled estimates in the abstract and conclusions.

**Response:** We agree this was the central weakness of the previous version and have revised the manuscript throughout to resolve this tension. The abstract, results, and discussion now consistently frame pooled estimates as exploratory/hypothesis-generating rather than confirmatory. Specifically: the abstract now states that findings ‘should be interpreted as exploratory evidence’ rather than confirmatory evidence of effectiveness; the overall tone of the discussion has been downgraded accordingly; and we have added explicit statements that interpretation is constrained by the predominance of low-quality reviews. We also reconciled the AMSTAR-2 quality figures so that the 95% (40/42) low-or-critically-low figure is used consistently across the abstract, results, and discussion.

**Comment 2 (Small pooled estimates):** Several pooled estimates are derived from only two or three reviews (e.g., self-management, distress, blood pressure), making them statistically unstable. The authors acknowledge this in the limitations but continue to present these findings as robust in the results and discussion. Forest plots should clearly annotate the small number of contributing reviews.

**Response:** All pooled estimates derived from only two contributing reviews (k=2), including blood pressure and self-management, have been removed from the meta-analyses; these outcomes are now reported narratively only, and the associated discussion of them as pooled findings has been deleted. Distress and related psychological wellbeing outcomes were likewise not pooled statistically due to small sample sizes and heterogeneity. For the remaining outcomes pooled from only three reviews (HbA1c and anxiety), we have retained the pooled estimate, as three reviews meets our a priori minimum for pooling, but we have added explicit annotation to both the figure captions and the accompanying text (‘Pooled effect estimated from 3 reviews’) so that the limited evidence base is transparent to the reader, in line with your recommendation.

**Comment 3 (Figure 7):** Figure 7 is referenced but missing from the submitted file.

**Response:** The reference to Figure 7 has been removed; no content in the manuscript now refers to a Figure 7 that does not exist. Figures have been renumbered sequentially to reflect the outcomes that remain pooled (Figures 1–6).

**Comment 4 (Abstract cut off):** The abstract is cut off mid-sentence in the PDF.

**Response:** We have confirmed that this was a PDF conversion/production error in the original submission and not a defect in the manuscript itself; the complete 209-word abstract has been present throughout. We have re-verified the PDF conversion for this resubmission to ensure the abstract renders in full.

**Comment 5 (Subgroup analyses):** Subgroup analyses are underutilized, no analysis by intervention modality (mHealth vs. telehealth vs. messaging) was performed despite substantial heterogeneity.

**Response:** We attempted subgroup analysis by intervention modality but found an insufficient number of reviews within each modality subgroup to produce statistically meaningful estimates; this is now stated explicitly in both the Discussion and the Strengths and Limitations section. We have retained the subgroup analyses that were feasible (age group and condition, for medication adherence and quality of life), which are reported in Supplemental Material Tables 1–3.

**Comment 6 (Repetitive discussion / generic future research):** The discussion is repetitive, restating the same limitations multiple times, and the future research section is generic.

**Response:** We have substantially edited the Discussion to remove repeated statements of the same limitation (particularly duplicated statements regarding AMSTAR-2 quality and small review counts), consolidating this material into a single Strengths and Limitations subsection. The Future Research section has been rewritten to be more specific, now identifying concrete gaps (patient activation, satisfaction, and health knowledge outcomes; longer-term primary studies; equity considerations for communicable diseases and low- and middle-income settings; and stratified analyses by individual DHI modality) rather than generic recommendations.

**Comment 7 (Minor issues):** Minor issues include inconsistent terminology (‘meta-meta-analysis’ vs. ‘umbrella review’), awkward phrasing, and occasional typographical errors.

**Response:** Terminology has been standardized throughout the manuscript: the paper is now consistently referred to as an ‘umbrella review,’ including in the title, and ‘meta-meta-analysis’ has been removed. We have also proofread the manuscript to correct awkward phrasing and typographical errors identified during revision (e.g., harmonizing UK/US spelling to US English throughout, per journal convention).

**E. Reviewer Recommendation**

**Reviewer #2:** Overall, the review is well-conducted and timely. However, the disconnect between the poor quality of the underlying evidence and the confident presentation of pooled estimates must be resolved. I recommend Revision.

**Response:** We thank Reviewer #2 for this assessment and hope that the revisions summarized above, particularly the consistent reframing of all pooled findings as exploratory, the removal of unstable k=2 pooled estimates, and the explicit annotation of contributing review counts, particularly those with 3 reviews, satisfactorily resolve this disconnect.
