# Supplementary material for: Digital health interventions for patient engagement: A systematic umbrella review
Source: PLOS Digit Health. 2026 Oct 30;5(10):e0001785. doi: 10.1371/journal.pdig.0001785 (PMC13722202; doi:10.1371/journal.pdig.0001785)
Supplement: Supplementary file 4 [file pdig.0001785.s007.docx]

**Response to Reviewers**

**A. Journal Requirements**

**Requirement 1:** While revising your submission, we strongly recommend that you use PLOS’s NAAS tool (https://ngplosjournals.pagemajik.ai/artanalysis) to test your figure files. NAAS can convert your figure files to the TIFF file type and meet basic requirements (such as print size, resolution) or provide you with a report on issues that do not meet our requirements and that NAAS cannot fix.

**Response:** All manuscript figures have been uploaded to the PLOS’s NAAS tool with all being passed or fixed without any failures.

**B. Additional Editor Comments**

**Editor:** This revision has genuinely resolved the substantive concern raised earlier about overclaiming from a low-quality evidence base. The remaining points are stylistic and easily fixed at copyedit or in a light final pass. I don't see anything here that should hold up publication. I'd support moving this forward once the authors do a final language pass on the caution and hedging language and reconcile the abstract-results wording on subgroup effects. Happy to sign off on acceptance after that light touch-up.

**Response:** We appreciate the editor's decision. We have quickly worked on the recommendations on using the PLOS’s NAAS tool for manuscript figures and additional suggestions from the reviewers including standardising tone of results, conducting additional corrected covered area calculations and clarifying criteria for statistical pooling. We hope these additional revisions will satisfy the requirements for the manuscript to proceed to the next step.

**C. Reviewer #1**

1. The authors have adequately addressed your comments raised in a previous round of review? Reviewer #1: All comments have been addressed
2. Does this manuscript meet PLOS Digital Health's publication criteria? Reviewer #1: Yes.
3. Has the statistical analysis been performed appropriately and rigorously? Reviewer #1: Yes.
4. Have the authors made all data underlying the findings fully available? Reviewer #1: Yes.
5. Is the manuscript presented in an intelligible fashion and written in standard English? Reviewer #1: Yes.

**Comments to the Author: Reviewer #**1: This paper thoroughly explains all of the needed information.

**Response:** We thank Reviewer #1 for their positive assessment of the manuscript's methodological soundness, statistical analysis, data availability, and clarity.

**D. Reviewer #2**

1. 1. The authors have adequately addressed your comments raised in a previous round of review? Reviewer #2: All comments have been addressed
2. Does this manuscript meet PLOS Digital Health's publication criteria? Reviewer #2: Partly.
3. Has the statistical analysis been performed appropriately and rigorously? Reviewer #2: No.
4. Have the authors made all data underlying the findings fully available? Reviewer #2: Yes.
5. Is the manuscript presented in an intelligible fashion and written in standard English? Reviewer #2: Yes.

**Comments to the Author: Reviewer #2:** The authors have made a serious effort to address the concerns raised in the previous round. The manuscript now appropriately presents the pooled findings as exploratory, removes analyses based on only two reviews, and is more transparent about the limited number of reviews contributing to several estimates. These revisions have improved the paper substantially.

**Comment 1 (Overlap among the reviews contributing to each pooled estimate):** The overall corrected covered area is low, but this does not establish that overlap is low within the small groups of reviews used for each meta-analysis. This is particularly important for outcomes based on only three or four reviews. Please report overlap for each pooled outcome and explain how duplicated primary studies were handled.

**Response:** Outcome specific corrected covered areas have been calculated for all quantitative outcomes with CCAs ranging from 0 to 2.7%, which allows for maintaining included reviews. A statement is included before description of meta-analysis results on page 9.

**Comment 2 (Interpretation of evidence from low-quality reviews):** All included reviews were rated low or critically low using AMSTAR-2. The revised manuscript acknowledges this, but several statements still imply effectiveness more strongly than the evidence permits. Language such as “produced improvements” and “supported the benefits” should be replaced consistently with more cautious wording. The comparison of the HbA1c estimate with clinically meaningful thresholds should also be removed or substantially qualified.

**Response:** HbA1c comparison with ADA thresholds has been removed. Statements mentioning “improvements” have been either removed or replaced with statistical language e.g. “estimates rather than improvements” or language suggesting the potential for DHIs.

**Comment 3 (Clinical heterogeneity):** The pooled analyses combine markedly different interventions, populations, outcome definitions, and measurement approaches. The manuscript should explain more clearly how reviews were judged sufficiently comparable for pooling. A concise table describing the interventions, outcome measures, follow-up periods, and populations contributing to each pooled estimate would help readers judge the validity of these analyses.

**Response:** The comment offers crucial insight. Criteria for pooling studies has been described in the third paragraph on page 6. Conceptually, PE being a process, allows for intervention combination, while specifying the outcome and outcome measures used. Statistically, use of random-effects considers variability around the effect estimate. Sub-group analyses were planned but not all were done which is also reported. S2 File includes all data (outcomes, specific interventions, population group, estimates, sample size and study periods) used in the meta-analysis. Table 1 in the manuscript offers a breakdown of intervention groups reported in included reviews.

**Comment 4 (Funnel Plot feasibility):** Funnel plots based on four or six reviews cannot meaningfully exclude publication bias. Please state that publication bias could not be reliably assessed.

**Response:** This is a truly insightful comment. Funnel plots have been removed from the supplementary material. This is also reflected in results section of the manuscript.

**Comment 5 (Inconsistent reporting on subgroup analysis):** The abstract says subgroup effects were comparable, whereas the Results report significant variation. These statements should be reconciled.

**Response:** This has been corrected by changing the statement in the abstract

**Comment 6 (Grammar):** The manuscript would benefit from another careful language edit.

**Response:** Careful revision of grammar and statement flow has been done.

**Comment 7 (Minor issues):** Minor issues include inconsistent terminology (‘meta-meta-analysis’ vs. ‘umbrella review’), awkward phrasing, and occasional typographical errors.

**Response:** Terminology has been standardized throughout the manuscript: the paper is now consistently referred to as an ‘umbrella review,’ including in the title, and ‘meta-meta-analysis’ has been removed. We have also proofread the manuscript to correct awkward phrasing and typographical errors identified during revision (e.g., harmonizing UK/US spelling to US English throughout, per journal convention).

**E. Reviewer Recommendation**

**Reviewer #2:** The revision is much improved, but the issues above should be resolved before acceptance.

**Response:** We thank Reviewer #2 for this assessment and hope that the additional revisions are satisfactory.
