# Supplementary material for: Digital health interventions for patient engagement: A systematic umbrella review
Source: PLOS Digit Health. 2026 Oct 30;5(10):e0001785. doi: 10.1371/journal.pdig.0001785 (PMC13722202; doi:10.1371/journal.pdig.0001785)
Supplement: Supplementary file 5 [file pdig.0001785.s008.docx]

**Response to Reviewers**

**A. Journal Requirements**

**Figure resubmission:** While revising your submission, we strongly recommend that you use PLOS’s NAAS tool (https://ngplosjournals.pagemajik.ai/artanalysis) to test your figure files. NAAS can convert your figure files to the TIFF file type and meet basic requirements (such as print size, resolution) or provide you with a report on issues that do not meet our requirements and that NAAS cannot fix.

**Response:** All manuscript figures were previously uploaded to the PLOS’s NAAS tool with all being passed or fixed without any failures.

**B. Reviewer #1**

1. The authors have adequately addressed your comments raised in a previous round of review? Reviewer #1: All comments have been addressed
2. Does this manuscript meet PLOS Digital Health's publication criteria? Reviewer #1: Partly.
3. Has the statistical analysis been performed appropriately and rigorously? Reviewer #1: I don't know.
4. Have the authors made all data underlying the findings fully available? Reviewer #1: (No Response).
5. Is the manuscript presented in an intelligible fashion and written in standard English? Reviewer #1: (No Response).

**Comments to the Author: Reviewer #**1

**Comment 1:** I have, however, noticed one remaining inconsistency that should be corrected. The Methods state that funnel plots were visually inspected to assess publication bias, whereas the Results state that publication-bias assessments were not conducted because fewer than ten studies contributed to each meta-analysis. These two statements cannot stand both in the final manuscript. Given the authors' response to the previous review, I assume that the Methods sentence is residual text and should simply be removed or revised.

**Response:** We thank Reviewer #1 for their positive assessment of the manuscript's revisions. In the first paragraph of page 5, we have revised the statement on publication bias to conform to results section.

**Comment 2:** I also think the description of the criteria used to determine whether reviews were sufficiently comparable for pooling could be made slightly more precise. The manuscript currently refers to pooling at least three compatible reviews with similar outcome measures. This is reasonable in principle, but “compatible” remains somewhat subjective given the considerable variation in populations, interventions, follow-up periods, and outcome definitions represented in the included reviews. I do not think another analysis is required, particularly since the supplementary material appears to provide the relevant review-level information, but one or two sentences clarifying the clinical criteria used before statistical pooling would improve methodological transparency.

**Response:** In the first paragraph of page 5, compatibility has been described based on similar outcome and outcome measures reported e.g. studies reporting SMDs for adherence were pooled together.

**Comment 3:** There are also a few minor presentation issues that should be addressed during the final revision. For example, the caption for Figure 5 refers to a mean difference although the reported effect measure is a standardized mean difference. There are also a small number of typographical and stylistic inconsistencies in the figure captions and text. A final careful proofreading pass should be sufficient to resolve these.

**Response:** We thank Reviewer #1 for this observation. Changes to caption under Figure 5 has been implemented. We have also made a check on all other figures and tables.

**Comment 4:** Overall, the manuscript is substantially stronger than the version previously reviewed. The major methodological and interpretive concerns have, in my view, been adequately addressed, and I do not see a need for another substantial round of revision. The remaining points are limited and readily correctable. Subject to these minor amendments, I would support publication.

**Response:** We thank Reviewer #1 for this encouragement.

**C. Reviewer #2**

1. 1. The authors have adequately addressed your comments raised in a previous round of review? Reviewer #2: All comments have been addressed
2. Does this manuscript meet PLOS Digital Health's publication criteria? Reviewer #2: Yes.
3. Has the statistical analysis been performed appropriately and rigorously? Reviewer #2: Yes.
4. Have the authors made all data underlying the findings fully available? Reviewer #2: Yes.
5. Is the manuscript presented in an intelligible fashion and written in standard English? Reviewer #2: Yes.

**Comments to the Author: Reviewer #2:**  Thank you for the careful and comprehensive revision. The manuscript has improved substantially, and the major concerns raised during the previous review have been satisfactorily addressed. In particular, the authors have strengthened the assessment of overlap between reviews, clarified the criteria for pooling, appropriately limited quantitative synthesis where evidence was insufficient, and substantially tempered the interpretation of findings given the predominantly low-quality evidence base. The reporting of subgroup analyses, limitations and uncertainty is also clearer, and the supplementary materials provide adequate transparency regarding the underlying data and statistical analysis. I have no further substantive comments. A few remaining minor language or internal consistency issues can be addressed during the editorial and copy-editing process. I consider the manuscript suitable for publication.

**Response:** We are thankful to Reviewer #3 for this insight. We have thoroughly gone through the document and corrected any typos or grammatical errors we could find.
