# Supplementary material for: Digital health interventions for patient engagement: A systematic umbrella review
Source: PLOS Digit Health. 2026 Oct 30;5(10):e0001785. doi: 10.1371/journal.pdig.0001785 (PMC13722202; doi:10.1371/journal.pdig.0001785)
Supplement: Peer review history

# Peer review: Decision Letter 0

**DOI:** 10.1371/journal.pdig.0001785.r001

**Authors:** Baki Kocaballi, Milit Patel

19 Jul 2026

Response to Reviewers Revised Manuscript with Track Changes Manuscript **Journal Requirements:**

1. Please provide separate figure files in .tif or .eps format.

For more information about figure files please see our guidelines:  LINK

https://journals.plos.org/digitalhealth/s/figures

https://journals.plos.org/digitalhealth/s/figures#loc-file-requirements

2. Please provide an Author Summary. This should appear in your manuscript between the Abstract (if applicable) and the Introduction, and should be 150–200 words long. The aim should be to make your findings accessible to a wide audience that includes both scientists and non-scientists. Sample summaries can be found on our website under Submission Guidelines: [LINK]

https://journals.plos.org/digitalhealth/s/submission-guidelines#loc-parts-of-a-submission

3. We have noticed that you have uploaded Supporting Information files, but you have not included a list of legends. Please add a full list of legends for your Supporting Information files after the references list.

4. As required by our policy on Data Availability, please ensure your manuscript or supplementary information includes the following:

A numbered table of all studies identified in the literature search, including those that were excluded from the analyses.

For every excluded study, the table should list the reason(s) for exclusion.

If any of the included studies are unpublished, include a link (URL) to the primary source or detailed information about how the content can be accessed.

A table of all data extracted from the primary research sources for the systematic review and/or meta-analysis. The table must include the following information for each study:

Name of data extractors and date of data extraction

Confirmation that the study was eligible to be included in the review.

All data extracted from each study for the reported systematic review and/or meta-analysis that would be needed to replicate your analyses.

If data or supporting information were obtained from another source (e.g. correspondence with the author of the original research article), please provide the source of data and dates on which the data/information were obtained by your research group.

If applicable for your analysis, a table showing the completed risk of bias and quality/certainty assessments for each study or outcome.  Please ensure this is provided for each domain or parameter assessed. For example, if you used the Cochrane risk-of-bias tool for randomized trials, provide answers to each of the signalling questions for each study. If you used GRADE to assess certainty of evidence, provide judgements about each of the quality of evidence factor. This should be provided for each outcome.

An explanation of how missing data were handled.

This information can be included in the main text, supplementary information, or relevant data repository. Please note that providing these underlying data is a requirement for publication in this journal, and if these data are not provided your manuscript might be rejected.

5. Please note that your Data Availability Statement is currently missing the repository name and the DOI/accession number of each dataset OR a direct link to access each database. If your manuscript is accepted for publication, you will be asked to provide these details on a very short timeline. We therefore suggest that you provide this information now, though we will not hold up the peer review process if you are unable.

**Additional Editor Comments (if provided):**

The two existing reviews split sharply: one recommends Accept with minimal comment, the other flags the same AMSTAR-2/interpretation problem I'm raising here and recommends Major Revision. Given the size of that gap, and that the more critical review is also the more substantive one, I'd weight it heavily in the decision. The Accept review doesn't engage with the quality-of-evidence issue at all, which is the paper's central weakness.

**Reviewers' Comments:**

**Comments to the Author**

1. Does this manuscript meet PLOS Digital Health’s publication criteria?

Reviewer #1: Yes

Reviewer #2: Partly

**********

2. Has the statistical analysis been performed appropriately and rigorously?

Reviewer #1: Yes

Reviewer #2: Yes

**********

3. Have the authors made all data underlying the findings in their manuscript fully available (please refer to the Data Availability Statement at the start of the manuscript PDF file)?

The PLOS Data policy

Reviewer #1: Yes

Reviewer #2: Yes

**********

4. Is the manuscript presented in an intelligible fashion and written in standard English?

Reviewer #1: Yes

Reviewer #2: Yes

**********

Reviewer #1: 1. After reviewing PLOS Digital Health's publication criteria, this paper does fulfill the criteria.

2. The statistical analysis has reasoning behind the incorporation of the analysis into the paper.

3. The data and supplemental material is available.

4. It is understandable.

Reviewer #2: This manuscript presents a systematic umbrella review and meta-meta-analysis of digital health interventions for patient engagement, synthesizing 42 systematic reviews (853 primary studies; >574,000 participants). The review is methodologically sound, prospectively registered, and follows PRISMA guidance. Pooled analyses show significant improvements in medication adherence (SMD = 0.65) and modest benefits for quality of life (SMD = 0.26) and HbA1c (MD = -0.45%).

The primary concern is a fundamental interpretational problem. The authors report that 40 of 42 included reviews (95%) were rated low or critically low quality using AMSTAR-2. Despite this, the abstract and conclusions present pooled estimates as though they carry confirmatory weight. The authors acknowledge this limitation but never resolve the tension—the manuscript cannot simultaneously claim that the evidence is severely constrained while presenting effect sizes as reliable. The authors should either reframe the entire paper as a methodological cautionary tale emphasizing the poor quality of the evidence base, or substantially downgrade the prominence of pooled estimates in the abstract and conclusions.

Several pooled estimates are derived from only two or three reviews (e.g., self-management, distress, blood pressure), making them statistically unstable. The authors acknowledge this in the limitations but continue to present these findings as robust in the results and discussion. Forest plots should clearly annotate the small number of contributing reviews.

Production issues: Figure 7 is referenced but missing from the submitted file. The abstract is cut off mid-sentence in the PDF. Subgroup analyses are underutilized—no analysis by intervention modality (mHealth vs. telehealth vs. messaging) was performed despite substantial heterogeneity. The discussion is repetitive, restating the same limitations multiple times, and the future research section is generic. Minor issues include inconsistent terminology ("meta-meta-analysis" vs. "umbrella review"), awkward phrasing, and occasional typographical errors.

Overall, the review is well-conducted and timely. However, the disconnect between the poor quality of the underlying evidence and the confident presentation of pooled estimates must be resolved. I recommend Revision.

**********

what does this mean?). If published, this will include your full peer review and any attached files.

**Do you want your identity to be public for this peer review?**  If you choose “no”, your identity will remain anonymous but your review may still be made public.

For information about this choice, including consent withdrawal, please see our Privacy Policy

Reviewer #1: **Yes:**  Aryan Bhattacharya

Reviewer #2: **Yes:**  Aasim Ayaz Wani

**********

**Figure resubmission:**

**Reproducibility:**

To enhance the reproducibility of your results, we recommend that authors of applicable studies deposit laboratory protocols in protocols.io, where a protocol can be assigned its own identifier (DOI) such that it can be cited independently in the future. Additionally, PLOS ONE offers an option to publish peer-reviewed clinical study protocols. Read more information on sharing protocols at https://plos.org/protocols?utm_medium=editorial-email&utm_source=authorletters&utm_campaign=protocols

# Author comment: Author response to Decision Letter 1

**DOI:** 10.1371/journal.pdig.0001785.r002

23 Jul 2026

Attachment Submitted filename: Response to editorial & reviewer comments_July2026.docx

# Peer review: Decision Letter 1

**DOI:** 10.1371/journal.pdig.0001785.r003

**Authors:** Baki Kocaballi, Milit Patel

4 Aug 2026

Response to ReviewersRevised Manuscript with Track ChangesManuscript**Journal Requirements:** **Additional Editor Comments (if provided):** **Reviewers' Comments:**

**Comments to the Author**

Reviewer #1: All comments have been addressed

Reviewer #2: All comments have been addressed

**********

publication criteria?

Reviewer #1: Yes

Reviewer #2: Partly

**********

3. Has the statistical analysis been performed appropriately and rigorously?

Reviewer #1: Yes

Reviewer #2: No

**********

4. Have the authors made all data underlying the findings in their manuscript fully available (please refer to the Data Availability Statement at the start of the manuscript PDF file)?

The PLOS Data policy

Reviewer #1: Yes

Reviewer #2: Yes

**********

5. Is the manuscript presented in an intelligible fashion and written in standard English?

Reviewer #1: Yes

Reviewer #2: Yes

**********

**Review Comments to the Author**

Reviewer #1: This paper thoroughly explains all of the needed information.

Reviewer #2: The authors have made a serious effort to address the concerns raised in the previous round. The manuscript now appropriately presents the pooled findings as exploratory, removes analyses based on only two reviews, and is more transparent about the limited number of reviews contributing to several estimates. These revisions have improved the paper substantially.

I have three remaining concerns.

1. Overlap among the reviews contributing to each pooled estimate

The overall corrected covered area is low, but this does not establish that overlap is low within the small groups of reviews used for each meta-analysis. This is particularly important for outcomes based on only three or four reviews. Please report overlap for each pooled outcome and explain how duplicated primary studies were handled.

2. Interpretation of evidence from low-quality reviews

All included reviews were rated low or critically low using AMSTAR-2. The revised manuscript acknowledges this, but several statements still imply effectiveness more strongly than the evidence permits. Language such as “produced improvements” and “supported the benefits” should be replaced consistently with more cautious wording. The comparison of the HbA1c estimate with clinically meaningful thresholds should also be removed or substantially qualified.

3. Clinical heterogeneity

The pooled analyses combine markedly different interventions, populations, outcome definitions, and measurement approaches. The manuscript should explain more clearly how reviews were judged sufficiently comparable for pooling. A concise table describing the interventions, outcome measures, follow-up periods, and populations contributing to each pooled estimate would help readers judge the validity of these analyses.

Minor points:

Funnel plots based on four or six reviews cannot meaningfully exclude publication bias. Please state that publication bias could not be reliably assessed.

The abstract says subgroup effects were comparable, whereas the Results report significant variation. These statements should be reconciled.

The manuscript would benefit from another careful language edit.

The revision is much improved, but the issues above should be resolved before acceptance.

**********

what does this mean?). If published, this will include your full peer review and any attached files.

**Do you want your identity to be public for this peer review?**  If you choose “no”, your identity will remain anonymous but your review may still be made public.

For information about this choice, including consent withdrawal, please see our Privacy Policy

Reviewer #1: **Yes:**  Aryan Bhattacharya

Reviewer #2: **Yes:**  Aasim Ayaz Wani

**********

**Figure resubmission:**

**Reproducibility:**

To enhance the reproducibility of your results, we recommend that authors of applicable studies deposit laboratory protocols in protocols.io, where a protocol can be assigned its own identifier (DOI) such that it can be cited independently in the future. Additionally, PLOS ONE offers an option to publish peer-reviewed clinical study protocols. Read more information on sharing protocols at https://plos.org/protocols?utm_medium=editorial-email&utm_source=authorletters&utm_campaign=protocols

# Author comment: Author response to Decision Letter 2

**DOI:** 10.1371/journal.pdig.0001785.r004

10 Aug 2026

Attachment Submitted filename: Response to editorial & reviewer comments_Aug2026.docx

# Peer review: Decision Letter 2

**DOI:** 10.1371/journal.pdig.0001785.r005

**Authors:** Baki Kocaballi

23 Sep 2026

Response to ReviewersRevised Manuscript with Track ChangesManuscript**Journal Requirements:** **Additional Editor Comments (if provided):** **Reviewers' Comments:**

**Comments to the Author**

Reviewer #2: All comments have been addressed

Reviewer #3: All comments have been addressed

**********

publication criteria?

Reviewer #2: Partly

Reviewer #3: Yes

**********

3. Has the statistical analysis been performed appropriately and rigorously?

Reviewer #2: I don't know

Reviewer #3: Yes

**********

4. Have the authors made all data underlying the findings in their manuscript fully available (please refer to the Data Availability Statement at the start of the manuscript PDF file)?

The PLOS Data policy

Reviewer #2: (No Response)

Reviewer #3: Yes

**********

5. Is the manuscript presented in an intelligible fashion and written in standard English?

Reviewer #2: (No Response)

Reviewer #3: Yes

**********

Reviewer #2: The manuscript has improved considerably in this revision, and the authors have made a serious effort to address the concerns raised in the previous round. In particular, the interpretation of the findings is now more appropriately calibrated to the quality of the underlying evidence. The limitations associated with the predominance of low-quality reviews, the small number of reviews contributing to several pooled estimates, and the considerable heterogeneity in some analyses are now stated much more clearly. The additional outcome-specific overlap calculations are also useful and help address the previous concern regarding duplication of primary studies across reviews.

The revised manuscript is also more transparent about what could and could not reasonably be pooled. I appreciate the decision not to statistically combine outcomes for which the available evidence was too limited or heterogeneous. The discussion of patient activation, self-management, and several mental-health and disease-control outcomes is consequently more defensible than in the previous version. Similarly, the more cautious framing of the pooled findings as exploratory evidence rather than definitive proof of effectiveness is appropriate.

I have, however, noticed one remaining inconsistency that should be corrected. The Methods state that funnel plots were visually inspected to assess publication bias, whereas the Results state that publication-bias assessments were not conducted because fewer than ten studies contributed to each meta-analysis. These two statements cannot both stand in the final manuscript. Given the authors' response to the previous review, I assume that the Methods sentence is residual text and should simply be removed or revised.

I also think the description of the criteria used to determine whether reviews were sufficiently comparable for pooling could be made slightly more precise. The manuscript currently refers to pooling at least three compatible reviews with similar outcome measures. This is reasonable in principle, but “compatible” remains somewhat subjective given the considerable variation in populations, interventions, follow-up periods, and outcome definitions represented in the included reviews. I do not think another analysis is required, particularly since the supplementary material appears to provide the relevant review-level information, but one or two sentences clarifying the clinical criteria used before statistical pooling would improve methodological transparency.

There are also a few minor presentation issues that should be addressed during the final revision. For example, the caption for Figure 5 refers to a mean difference although the reported effect measure is a standardized mean difference. There are also a small number of typographical and stylistic inconsistencies in the figure captions and text. A final careful proofreading pass should be sufficient to resolve these.

Overall, the manuscript is substantially stronger than the version previously reviewed. The major methodological and interpretive concerns have, in my view, been adequately addressed, and I do not see a need for another substantial round of revision. The remaining points are limited and readily correctable. Subject to these minor amendments, I would support publication.

Reviewer #3: Thank you for the careful and comprehensive revision. The manuscript has improved substantially, and the major concerns raised during the previous review have been satisfactorily addressed. In particular, the authors have strengthened the assessment of overlap between reviews, clarified the criteria for pooling, appropriately limited quantitative synthesis where evidence was insufficient, and substantially tempered the interpretation of findings given the predominantly low-quality evidence base. The reporting of subgroup analyses, limitations and uncertainty is also clearer, and the supplementary materials provide adequate transparency regarding the underlying data and statistical analysis. I have no further substantive comments. A few remaining minor language or internal consistency issues can be addressed during the editorial and copy-editing process. I consider the manuscript suitable for publication.

**********

what does this mean?). If published, this will include your full peer review and any attached files.

**Do you want your identity to be public for this peer review?**  If you choose “no”, your identity will remain anonymous but your review may still be made public.

For information about this choice, including consent withdrawal, please see our Privacy Policy

Reviewer #2: No

Reviewer #3: **Yes:**  Dr K Madan Gopal

**********

**Figure resubmission:**

**Reproducibility:**

To enhance the reproducibility of your results, we recommend that authors of applicable studies deposit laboratory protocols in protocols.io, where a protocol can be assigned its own identifier (DOI) such that it can be cited independently in the future. Additionally, PLOS ONE offers an option to publish peer-reviewed clinical study protocols. Read more information on sharing protocols at https://plos.org/protocols?utm_medium=editorial-email&utm_source=authorletters&utm_campaign=protocols

# Author comment: Author response to Decision Letter 3

**DOI:** 10.1371/journal.pdig.0001785.r006

26 Sep 2026

Attachment Submitted filename: Response to editorial & reviewer comments_Sep2026.docx

# Editor report: Decision Letter 3

**DOI:** 10.1371/journal.pdig.0001785.r007

**Authors:** Baki Kocaballi

2 Oct 2026

Digital Health Interventions for Patient Engagement: A Systematic Umbrella Review

PDIG-D-26-00712R3

Dear Dr. Celi,

We are pleased to inform you that your manuscript 'Digital Health Interventions for Patient Engagement: A Systematic Umbrella Review' has been provisionally accepted for publication in PLOS Digital Health.

Before your manuscript can be formally accepted you will need to complete some formatting changes, which you will receive in a follow-up email from a member of our team.

Please note that your manuscript will not be scheduled for publication until you have made the required changes, so a swift response is appreciated.

IMPORTANT: The editorial review process is now complete. PLOS will only permit corrections to spelling, formatting or significant scientific errors from this point onwards. Requests for major changes, or any which affect the scientific understanding of your work, will cause delays to the publication date of your manuscript.

If your institution or institutions have a press office, please notify them about your upcoming paper to help maximize its impact. If they'll be preparing press materials, please inform our press team as soon as possible -- no later than 48 hours after receiving the formal acceptance. Your manuscript will remain under strict press embargo until 2 pm Eastern Time on the date of publication. For more information, please contact digitalhealth@plos.org.

Thank you again for supporting Open Access publishing; we are looking forward to publishing your work in PLOS Digital Health.

Best regards,

Baki Kocaballi

Section Editor

PLOS Digital Health

***********************************************************

**Additional Editor Comments (if provided):**

**Reviewer Comments (if any, and for reference):**
